# Serinc2 deficiency causes susceptibility to sepsis-associated acute lung injury

**DOI:** 10.1186/s12950-022-00306-x

**Published:** 2022-07-07

**Authors:** Shuai Mao, Jian Lv, Meng Chen, Ningning Guo, Yu Fang, Jingjing Tong, Xianghu He, Gang Wu, Zhihua Wang

**Affiliations:** 1 Department of Cardiology, Renmin Hospital of Wuhan University, Wuhan, 430061 China; 2 Shenzhen Key Laboratory of Cardiovascular Disease, Fuwai Hospital Chinese Academy of Medical Sciences, Shenzhen, Shenzhen, 518057 China; 3 State Key Laboratory of Cardiovascular Disease, National Center for Cardiovascular Diseases, Fuwai Hospital, Chinese Academy of Medical Sciences and Peking Union Medical College, Beijing, 100037 China; 4 Department of Anesthesiology, Zhongnan Hospital of Wuhan University, Wuhan, 430071 China; 5 School of Life Sciences, Central China Normal University, Wuhan, 430068 China

**Keywords:** Serinc2, Acute lung injury, Sepsis, Lipopolysaccharide, Inflammation, Apoptosis

## Abstract

**Background:**

Severe sepsis and its subsequent complications cause high morbidity and mortality rates worldwide. The lung is one of the most vulnerable organs sensitive to the sepsis-associated inflammatory storm and usually develops into acute respiratory distress syndrome (ARDS)/acute lung injury (ALI). The pathogenesis of sepsis-associated ALI is accompanied by coordinated transmembrane signal transduction and subsequent programmed cell death; however, the underlying mechanism remains largely unclear.

**Results:**

Here we find that the expression of serine incorporator 2 (Serinc2), a protein involved in phosphatidylserine synthesis and membrane incorporation, is upregulated in cecal ligation and puncture (CLP)-induced ALI. Furthermore, the Serinc2-knockout (KO) mouse line is generated by the CRISPR-cas9 approach. Compared with wild-type mice, the Serinc2-KO mice exhibit exacerbated ALI-related pathologies after CLP. The expressions of pro-inflammatory factors, including IL1β, IL6, TNFα, and MCP1, are significantly enhanced by Serinc2 deficiency, concurrent with over-activation of STAT3, p38 and ERK pathways. Conversely, Serinc2 overexpression in RAW264.7 cells significantly suppresses the inflammatory responses induced by lipopolysaccharide (LPS). Serinc2 KO aggravates CLP-induced apoptosis as evidenced by increases in TUNEL-positive staining, Bax expression, and cleaved caspase-3 and decreases in BCL-2 expression and Akt phosphorylation, whereas these changes are suppressed by Serinc2 overexpression in LPS-treated RAW264.7 cells. Moreover, the administration of AKTin, an inhibitor of Akt, abolishes the protective effects of Serinc2 overexpression against inflammation and apoptosis.

**Conclusions:**

Our findings demonstrate a protective role of Serinc2 in the lung through activating the Akt pathway, and provide novel insight into the pathogenesis of sepsis-induced ALI.

**Supplementary Information:**

The online version contains supplementary material available at 10.1186/s12950-022-00306-x.

## Introduction

Severe sepsis is a major healthcare problem that affects millions of patients globally each year. It causes devastating clinical illnesses, particularly acute respiratory distress syndrome (ARDS)/acute lung injury (ALI) [1]. The newly emerging COVID-19 pandemic also poses an increasing threat to humans with ARDS-related respiratory failure [2]. Sepsis and COVID-19-induced ARDS/ALI is characterized by large numbers of neutrophils, macrophages, and erythrocytes in the alveoli, hyaline membranes in the alveolar cavity, interstitial and alveolar edema, and necrosis of pulmonary endothelium and epithelium cells [1]. These pathologies are consequences of hyperactive host inflammatory responses to pathogens as evidenced by excessive release of chemokines and cytokines storm, such as IL1, IL6, CXCL2, TNFα, and MCP-1 [3–6].

Hyperactivation of the immune system during ALI triggers apoptosis of pulmonary vascular endothelial cells and alveolar epithelial cells, causing alveolar-capillary barrier damage, fluid leakage, and pulmonary hemorrhage [7–9]. In turn, the clearance of apoptotic cell corpse further enhances the inflammatory response, leading to a lethal cytokine storm [10]. How the inflammation and apoptosis pathways are coordinated during the pathogenesis of ALI remains largely unknown.

Serine incorporator 2 (Serinc2; also known as tumor differentially expressed protein 2, TDE2) belongs to the highly conserved Serinc family (1–5), which are responsible for serine synthesis and membrane incorporation [11]. It was first identified as an oncogene in non-small cell lung cancer by Player et al. in 2003 [12]. Zeng et al. [13] also found a high enrichment of Serinc2 in the lung adenocarcinoma that regulates tumor cell proliferation, migration, and invasion through the PI3K/AKT signaling pathway. However, the physiological function of Serinc2 in the lung remains unexplored.

Here, we generated a Serinc2-knockout (KO) mouse line and found that Serinc2-KO mice are vulnerable to cecal ligation and puncture (CLP)-induced ALI. Serinc2 deficiency exacerbated severe inflammation and apoptosis accompanied by excessive activation of multiple inflammation effectors, such as stat3, and P38/ERK. Moreover, Serinc2 overexpression in raw264.7 cells prevent lipopolysaccharide (LPS)-induced over-activation of inflammation and apoptosis through suppressing the STAT3 and P38 / ERK signaling pathways while activating the Akt signaling pathway. Our findings reveal a previously unrecognized protective role of Serinc2 in the pathogenesis of ALI and provide novel targets to treat infection-associated ALI in clinics.

## Materials and methods

### Generation of Serinc2-knockout mouse

CRISPR/Cas9 technology was used to delete the Serinc2 gene in the mouse by targeting exon 2–10 of the Serinc2 transcript (ENSMUST00000122374.7). Recombinant Cas9 protein and in vitro transcribed sgRNA were microinjected into the fertilized eggs of C57BL/6JGpt mice. Fertilized eggs were transplanted to obtain positive F0 mice which were confirmed by PCR and sequencing. A stable F1 generation mouse model was obtained by mating positive F0 generation mice with C57BL/6JGpt mice. Then we inter-crossed heterozygous females and males to obtain the third generation homozygous Serinc2^−/−^ mice for formal experiments.

### Animal care

All animal experiments procedures were reviewed and approved by the Institutional Animal Care and Use Committee (IACUC) of Renmin Hospital of Wuhan University and performed by the guide for the care and use of laboratory animals published by the National Institutes of Health, USA (8th edition). All mice were raised in a specific pathogen-free environment (temperature, 24 ± 3 °C; humidity, 55 ± 5%) with a 12-h light/12-h dark cycle and fed normal chow.

### Cecal ligation and puncture (CLP) surgery

Serinc2^−/−^ and wildtype (WT) C57BL/6 J mice were randomly assigned into sham or CLP groups. CLP surgery was performed as described [14]. Mice were anesthetized with 1% pentobarbital sodium (50 mg/kg) by intraperitoneal (i.p.) injection. After anesthesia, mice were fixed on the operation table, the abdominal hair was removed first, and a 1 midline cm incision was made along the abdominal white line. Then open the abdominal cavity layer by layer and find the cecum. the cecum was tightly ligated with 3 / 0 silk thread at 1 cm from the end of the cecum and punctured twice at 0.5 cm from the distal end with No.7 needle. Then gently squeeze to extrude a small number of feces. After replacing the cecum in the abdomen, the abdomen was closed in two layers. Mice in the sham group underwent the same procedure except their cecum were neither ligated nor punctured.

### Assessment of lung endothelial cell permeability

Lung endothelial cell permeability was detected by measuring the accumulation of Evans blue in the lungs. Evans blue (EB) dye solution (20 mg/kg) was injected into the tail vein 30 min before mice were sacrificed. Then lung tissues were perfused with PBS and collected. And the collected lung tissues were homogenized in a 37 ℃ bath for 24 h and centrifuged at 3000 g for 30 min. The EB content was determined by measuring the absorbance of the supernatant at 620 nm and corrected for hemoglobin content at 740 nm in the microplate reader according to the standard of known EB content.

### Hematoxylin and Eosin (H&E) staining

Mice were sacrificed 24 h after CLP. The lung tissues from part of the right middle lobe of the lung were collected and fixed with 4% paraformaldehyde at 25 ℃ for 24 h. After fixation, the lung tissues were embedded in paraffin with a slice thickness of 5 μm. H&E was stained with hematoxylin for 10 min and eosin for 3 min at 25℃. The degree of lung injury was graded using a histologic ALI scoring system based on histologic features, including neutrophils, hyaline membranes, proteinaceous debris, and alveolar septal thickening. All lung fields at × 20 magnification were examined for each sample. Assessment of histological lung injury was performed by scoring from 1 to 5, with 1 being the best (normal lung) and 5 being the worst (most severe ALI). Scoring was performed as follows: 1, normal; 2, focal (< 50% lung section) interstitial congestion and inflammatory cell infiltration; 3, diffuse (> 50% lung section) interstitial congestion and inflammatory cell infiltration; 4, focal (< 50% lung section) consolidation (combining into a solid mass without the alveoli structure) and inflammatory cell infiltration and 5, diffuse (> 50% lung section) consolidation and inflammatory cell infiltration [15, 16].

### Immunohistochemical staining

The wax blocks were prepared, sliced into 5 μm thick slices, dewaxed and hydrated, the endogenous peroxidase was eliminated by 3%H202 at room temperature for 10 min, washed with PBS, antigen repair at high temperature and high pressure, washed with PBS again, and dropped anti-Serinc2 primary antibody. After washing with PBS, the relative second antibody was incubated at room temperature for 30 min. After washing with PBS, DAB was used for color rendering, and water was rinsed for 10 min. Hematoxylin was restained, dehydrated, transparent, neutral adhesive was sealed, and observed under an optical microscope.

### Cell culture and treatment

RAW264.7 cells were cultured in DMEM containing 10% fetal bovine serum (FBS; Gibco, USA) and incubated at 37℃ in a humidified atmosphere containing 5% CO_2_. RAW264.7 cells were stimulated with LPS (Sigma, USA) to induce cellular damage as an in vitro ALI model. Plasmid pcDNA3.1-Serinc2-3 × flag (4 μg) and negative control pcDNA3.1-EGFP(4 μg) were transiently transfected into RAW264.7 cells using Lipofectamine 2000 (6 μl, Invitrogen, USA). Then, the cells were subjected to 1 μg/ml LPS with or without an AKT inhibitor, AKTin (1 μM). The cells were collected and analyzed for molecular measurements.

### Terminal deoxynucleotidyl transferase dUTP nick end labeling (TUNEL) staining

Apoptosis was detected and quantified by the TUNEL assay using the In Situ Cell Death Detection Kit according to manufacturer protocols (Sigma-Aldrich). Five representative fields were selected from each group. The number of TUNEL positive cells in 500 cells was counted and AI was calculated. AI = apoptosis-positive number/total number of cells × 100%.

### Quantitative real-time polymerase chain reaction (qRT-PCR)

Total RNA was extracted from cells or tissues using Trizol reagent (TaKaRa, Shiga, Japan). The RNA purity and concentration were determined according to the ratio 260/280 nm in a UV spectrophotometer. Total RNA was reverse transcribed into cDNA using the Revert Aid First Strand cDNA synthesis Kit (Thermo). qRT-PCR detection was subsequently performed using LightCyclerR 480 (Roche, Switzerland). The expression of Gapdh was used to normalize the gene expression levels. The primers of target genes were: IL-1β_F, CCGTGGACCTTCCAGGATGA; IL-1β_R, GGGAACGTCACACACCAGCA; IL-6_F, CTGCAAGAGACTTCCATCCAG; IL-6_R, AGTGGTATAGACAGGTCTGTTGG; TNF-α_F, CATCTTCTCAAAATTCGAGTGACAA; TNF-α_R, TGGGAGTAGACAAGGTACAACCC; MCP-1_F, TAAAAACCTGGATCGGAACCAAA; MCP-1_R, GCATTAGCTTCAGATTTACGGGT; GAPDH_F, ACCCTTAAGAGGGATGCTGC; GAPDH_R, CCCAATACGGCCAAATCCGT; Serinc2_F, GACTCTTTGTGTAACTGGCATC; Serinc2_R, AGCTTGTAAAGCTGACTCTCCA.

#### Western blot analysis

RIPA lysis buffer was used to extract protein from cells and frozen lung tissues. BCA protein quantitative kit was used to determine protein concentration. The same amount of protein (40 μg) in each sample of at least 3 biological replicates was separated by SDS-PAGE and then transferred to the PVDF membrane. After sealing these membranes with 5% skimmed milk powder at room temperature for 1 h, these membranes were incubated overnight with the corresponding primary antibodies against SERINC2 (1:1000, PA5-49,872, Invitrogen), p-STAT3 [1:1000, 9145, Cell Signaling Technology (CST)], STAT3 (1:1000, 12,640, CST), p-p38 (1:1000, 4511, CST), p38 (1:1000, 8690, CST), p-ERK (1:1000, 4370S, CST), p44/42MPAK (ERK1/2; 1:1000, 9102S, CST), p-AKT (1:1000, 4060S, CST), AKT (1:000,9272S, CST), GAPDH (1:1000, 5174, CST) at 4 ℃. Then, it was incubated with the horseradish peroxidase binding second antibody at room temperature for 1 h. After that, the target strip was exposed to the chemiluminescence imaging system with a chemiluminescence developer, and the target strip was quantitatively analyzed by Image J (National Institutes of Health). The GAPDH protein content in each sample was used as a reference.

### Enzyme-linked immunosorbent assay (ELISA)

Mice were sacrificed 24 h after CLP. the arterial blood was collected in heparin anticoagulant tubes and centrifuged. The average absorbance value was determined by the corresponding enzyme-linked immunosorbent assay (ELISA) kit, IL6 (Thermo Fisher, USA), TNFα (Thermo Fisher, USA). The absorbance of each well was recorded at 450 nm using a microplate reader.

### Bioinformatic analysis

Data files including GSE62107, GSE8608, GSE1871, GSE40885, and GSE45644 were downloaded from Gene Expression Omnibus (GEO) (http://www.ncbi.nlm.nih.gov/geo/). LPS stimulation was used to perform the ALI model in all these profiles. GSE62107 included 1 control sample and 1 LPS treatment sample, GSE8608 included 1 control sample and 1 LPS treatment sample, GSE1871 included 3 control samples and 3 LPS treatment samples, GSE40885 included 7 control samples and 7 LPS treatment samples, GSE45644 included 5 control samples and 5 LPS treatment samples.

### Statistical analyses

Statistical analyses were performed using GraphPad Prism 8 Software. All experimental data are presented as mean ± SEM of at least three independent experiments. Statistical significance for multiple comparisons was determined by one-way ANOVA or two-way ANOVA followed by Tukey’s test. Bonferroni adjustment was used for post hoc analysis. The student’s *t*-test was used for comparisons between two groups. *P* < 0.05 was considered statistically significant.

## Results

### Serinc2 deficiency exacerbates CLP-induced ALI

**Fig. 1:**
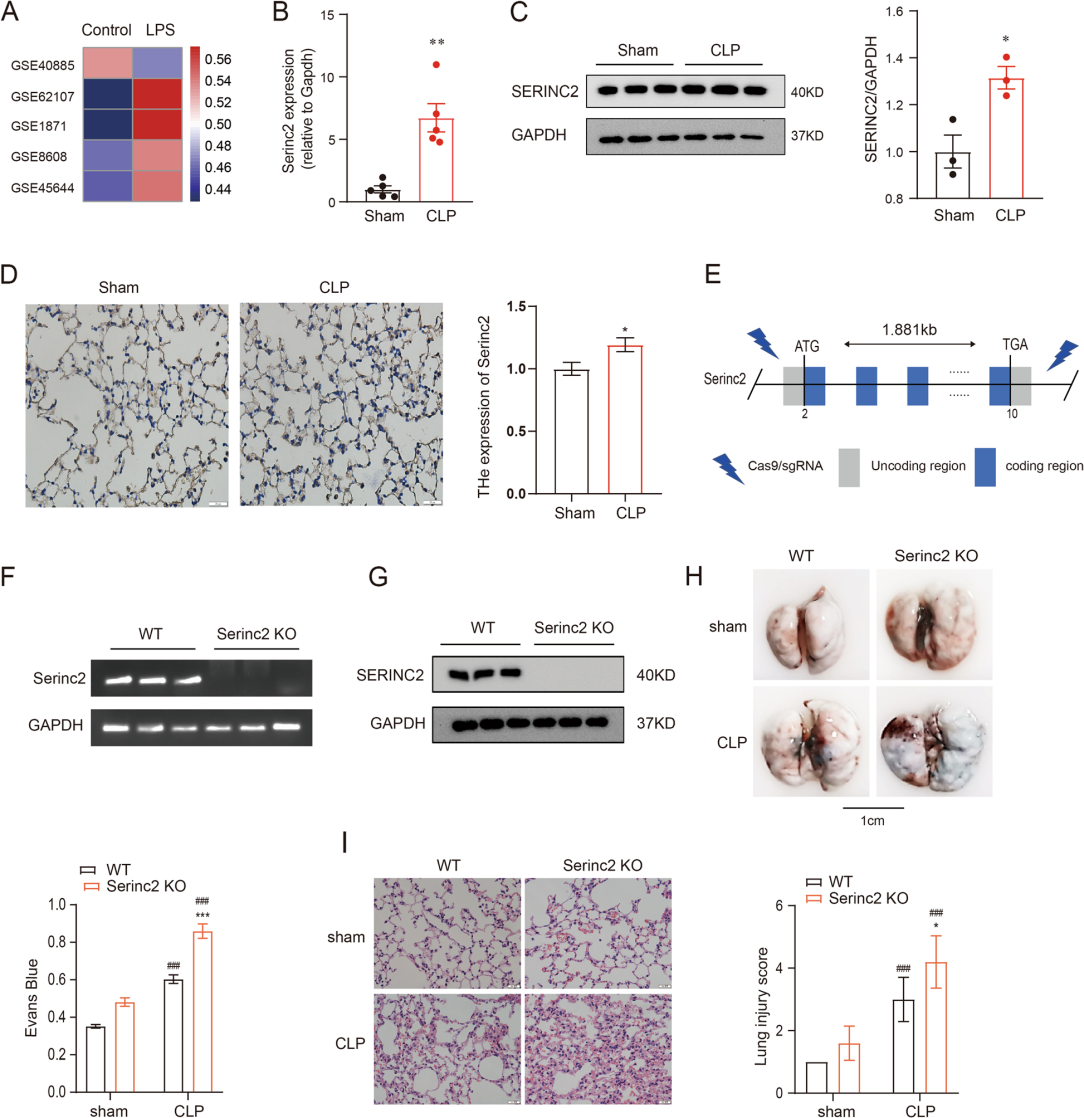
Serinc2 KO exacerbates CLP-induced ALI. **A** Bioinformatic analysis showing the regulation of Serinc2 expression after LPS stimulation from indicated GEO databases. **B** Effect of CLP on Serinc2 expression at the mRNA level in the lung measured by qRT-PCR. **p < 0.01 vs. sham; *n* = 5. **C** Immunoblots (left) and Quantification data (right) showing the expression of SERINC2 in the lung tissues of mice with sham or CLP surgeries. GADPH serves as the inner control. *p < 0.05 versus CLP alone. *p < 0.05 vs. sham; *n* = 5. **D** Immunohistochemical staining and corresponding statistical results showing the expression of Serinc2 in the lung tissue with or without CLP surgeries. **E** Schematic showing the strategy to generate Serinc2 knockout (Serinc2^−/−^) mouse using CRISPR/Cas9 technology. **F** Validation of Serinc2 knockout by agarose gel electrophoresis for the PCR products with Serinc2 (Exon2-10) primers. **G** Immunoblots showing the complete knockout of SERINC2 in the lung tissues. **H** Representative Evan’s blue images (left) and quantification data (right) showing the impact of Serinc2 knockout on alveolar capillary barrier damage induced by CLP. ***P < 0.001 vs. WT; ###*P* < 0.001 vs. sham. *n* = 5. **I** Representative H&E staining images and the ALI scoring for the H&E staining showing the impact of Serinc2 knockout on CLP-induced pathology. *n* = 5

Since Serinc2 has been functionally involved in normal lung function [12, 13], we firstly explored the regulation of Serinc2 in ALI by examining its expression in available transcriptome profiles of ALI models. The expression level of Serinc2 was upregulated in 4 out of 5 GEO databases with LPS treatment (Fig. 1A). We then performed CLP surgery to induce ALI and found that the expression of Serinc2 significantly increased in the lung after CLP surgery (Fig. 1B-D), suggesting a possible involvement of Serinc2 in the pathogenesis of CLP-induced ALI.

To investigate the physiological role of Serinc2, we generated a Serinc2-KO (Serinc2^−/−^) mouse line using the CRISPR/Cas9 system (Fig. 1E). Heterozygous Serinc2^±^ mice had normal fertility and gave birth to Serinc2^+/+^, Serinc2^±^, and Serinc2^−/−^ mice basically according to Mendel’s law as expected (Supplementary Table 1). Mating between male and female Serinc2^−/−^ mice also gave birth to normally developed Serinc2^−/−^ mice (Fig. 1F and G), suggesting that Serinc2 is dispensable for gametogenesis and development.

When these mice were subjected to CLP surgery, however, three out of 10 Serinc2^−/−^ mice died from CLP-induced sepsis within 24 h, while all WT mice survived. Serinc2 deficiency significantly increased the CLP-induced EB dye leakage (Fig. 1H). Moreover, Serinc2 deficiency exacerbated ALI pathologies, including increased lung septum, pulmonary vascular congestion, and inflammatory cell infiltration (Fig. 1I). Consistently, histologic ALI scoring in Serinc2^−/−^ mice was significantly elevated compared with WT after CLP (Fig. 1I). These data suggest that Serinc2 plays a protective role in CLP-induced ALI, while its upregulation in ALI might contribute as an endogenous protective feedback response.

### Serinc2 KO promotes inflammatory responses and activates STAT3, p38, and ERK pathways in CLP-induced ALI

**Fig. 2:**
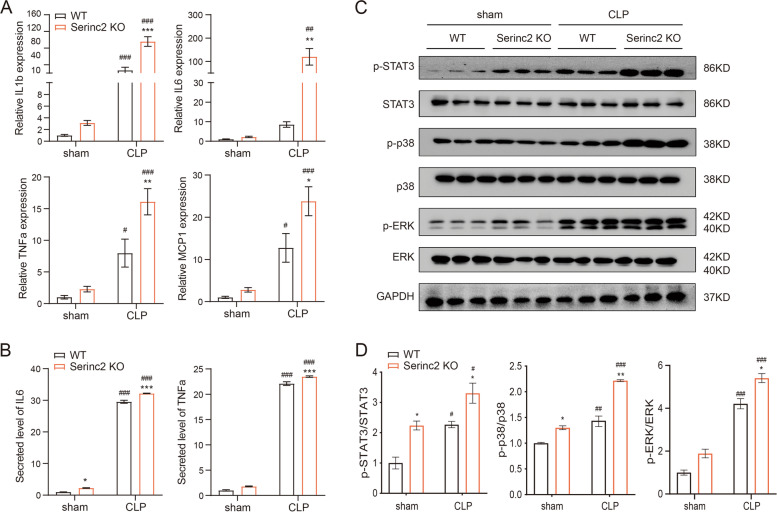
Serinc2 KO enhances CLP-induced hyperactivation of inflammation. **A** Impact of Serinc2 KO on CLP-induced expression of IL1β, IL6, TNFα, and MCP1 measured by qRT-PCR. *P < 0.05, **P < 0.01, ***P < 0.001 vs. WT; #*P* < 0.05, ##*P* < 0.01, ###*P* < 0.001 vs. sham. *n* = 5–7. **B** Impact of Serinc2 KO on CLP-induced secretion of IL6 and TNFα in serum assessed by ELISA kits. *P < 0.05, ***P < 0.001 vs. WT; ###*P* < 0.001 vs. sham. *n* = 5–7. **C** Immunoblots of phosphorylated and total STAT3, p38, and ERK in the lung tissues from WT and Serinc2-KO mice with sham or CLP surgeries. **D** Quantification of STAT3, p38 and ERK phosphorylation levels in ratio to their total protein. *P < 0.05, **P < 0.01 vs. WT; #*P *< 0.05, ##*P* < 0.01, ###*P* < 0.001 vs. sham. *n* = 3

Cytokines such as IL1β, IL6, TNFα, and MCP1 are involved in the inflammation process during ALI [3, 4]. We measured the expression of these cytokines in the lung by qRT-PCR. The results showed that Serinc2 deficiency-induced expression of IL1β, IL6, TNFα, and MCP1 at basal condition, and further enhanced their increased mRNA levels after CLP (Fig. 2A). Consistently, the ELISA assay detected significant increases in circulating IL6 and TNFα in the Serinc2-KO group compared with the WT controls both in sham and CLP groups (Fig. 2B), suggesting that Serinc2 deficiency augments the inflammatory responses in CLP-induced ALI.

Host inflammation responses in sepsis-associated ALI are mediated by pro-inflammatory signaling pathways, such as signal transducers and activators of transcription 3 (STAT3), extracellular signal-regulated kinase (ERK), p38, etc [17, 18]. Western blot analysis detected significant increases in phosphorylation of STAT3, p38, and ERK 24 h after CLP in WT mice, which were further enhanced in Serinc2-KO mice (Fig. 2C and D), suggesting that the impact of Serinc2 deficiency on inflammation is mediated by the hyperactivation of these pro-inflammatory pathways.

### Serinc2 overexpression ameliorates LPS-induced inflammation in RAW264.7 cells

**Fig. 3:**
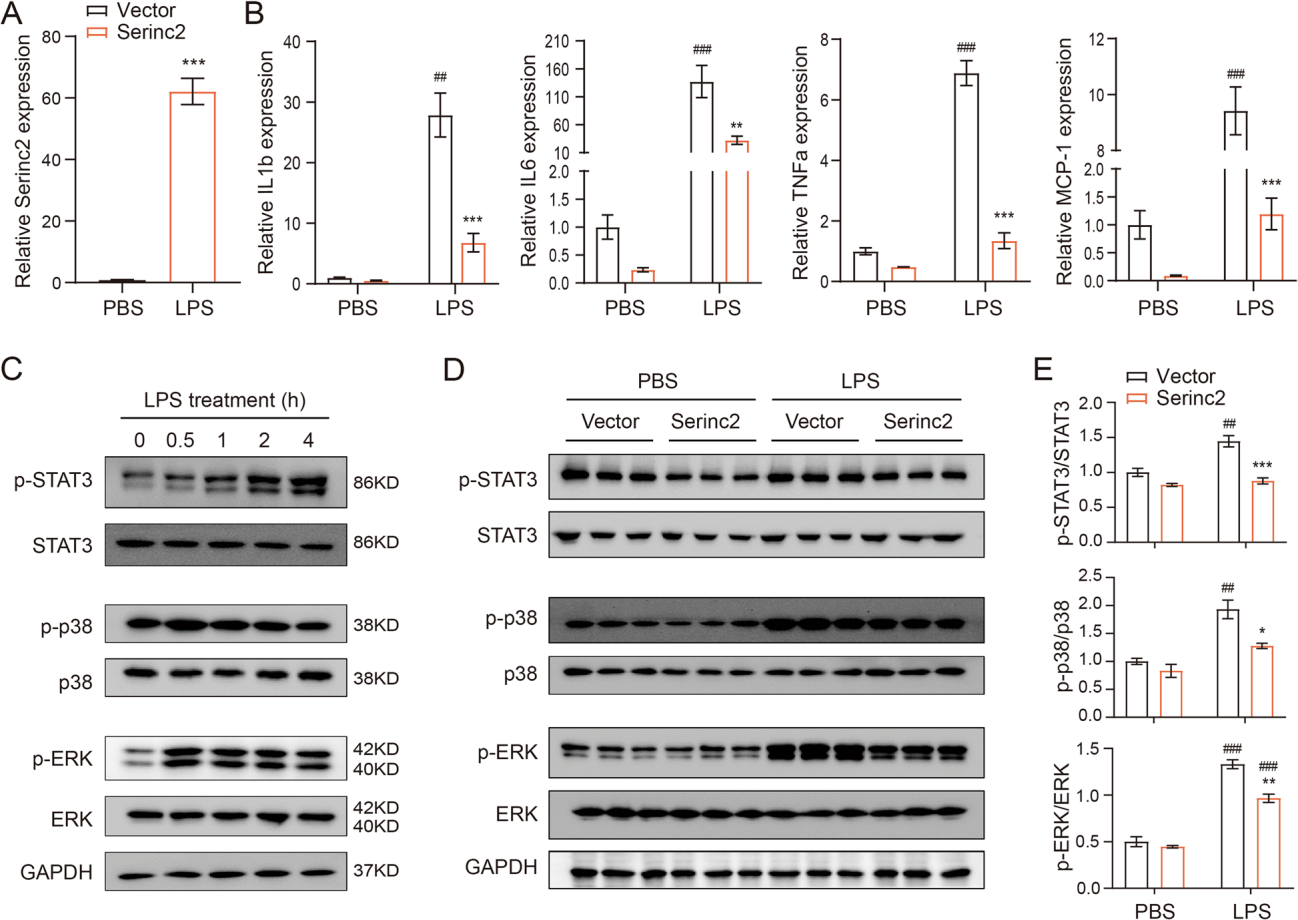
Serinc2 overexpression protects RAW264.7 cells from LPS-induced inflammation. **A** Validation of Serinc2 overexpression by qRT-PCR. RAW264.7 cells were transfected with Vector or Serinc2 plasmid (4 μg) for 48 h. ***P < 0.001 vs. Vector. *n* = 3. **B** Effects of Serinc2 overexpression on LPS-induced expression of IL1β, IL6, TNFα, and MCP1 in RAW264.7 cells. LPS (1 μg) was administrated for 4 h. **P < 0.01, ***P < 0.001 vs. Vector; ##*P* < 0.01, ###*P* < 0.001 vs. PBS. *n* = 3. **C** Time-dependent activation of STAT3, p38, and ERK pathways by LPS in RAW264.7 cells measured by Western blot. **D** Immunoblots showing the impact of Serinc2 overexpression on LPS-induced phosphorylation of STAT3, p38, and ERK. **E** Quantification of STAT3, p38 and ERK phosphorylation levels in ratio to their total protein. *P < 0.05, **P < 0.01, ***P < 0.001 vs. Vector; ##*P* < 0.01, ###*P* < 0.001 vs. PBS. *n* = 3

Macrophage plays a central role in the initiation, maintenance, and remission of inflammation during infection [19]. To determine the protective effect of Serinc2 in ALI, we overexpressed Serinc2 in LPS-stimulated RAW264.7 macrophages (Fig. 3A). At the basal level, Serinc2 overexpression reduced the expression of IL1β, IL6, TNFα, and MCP1 (Fig. 3B). LPS treatment (1 μg/ml) for 24 h induced the expression of IL1β, IL6, TNFα, and MCP1, which were significantly reversed by Serinc2 overexpression (Fig. 3B). These data suggest that Serinc2 overexpression is powerful to suppress inflammation in ALI.

Phosphorylated STAT3 was time-dependently increased after LPS treatment in RAW264.7 cells, whereas the phosphorylation ERK and p38 were immediately enhanced upon LPS treatment (0.5 h) (Fig. 3C). After Serinc2 overexpression, the LPS-activated STAT3, p38, and ERK were significantly inhibited (Fig. 3D and E).

### Serinc2 inhibits cell apoptosis in ALI

**Fig. 4:**
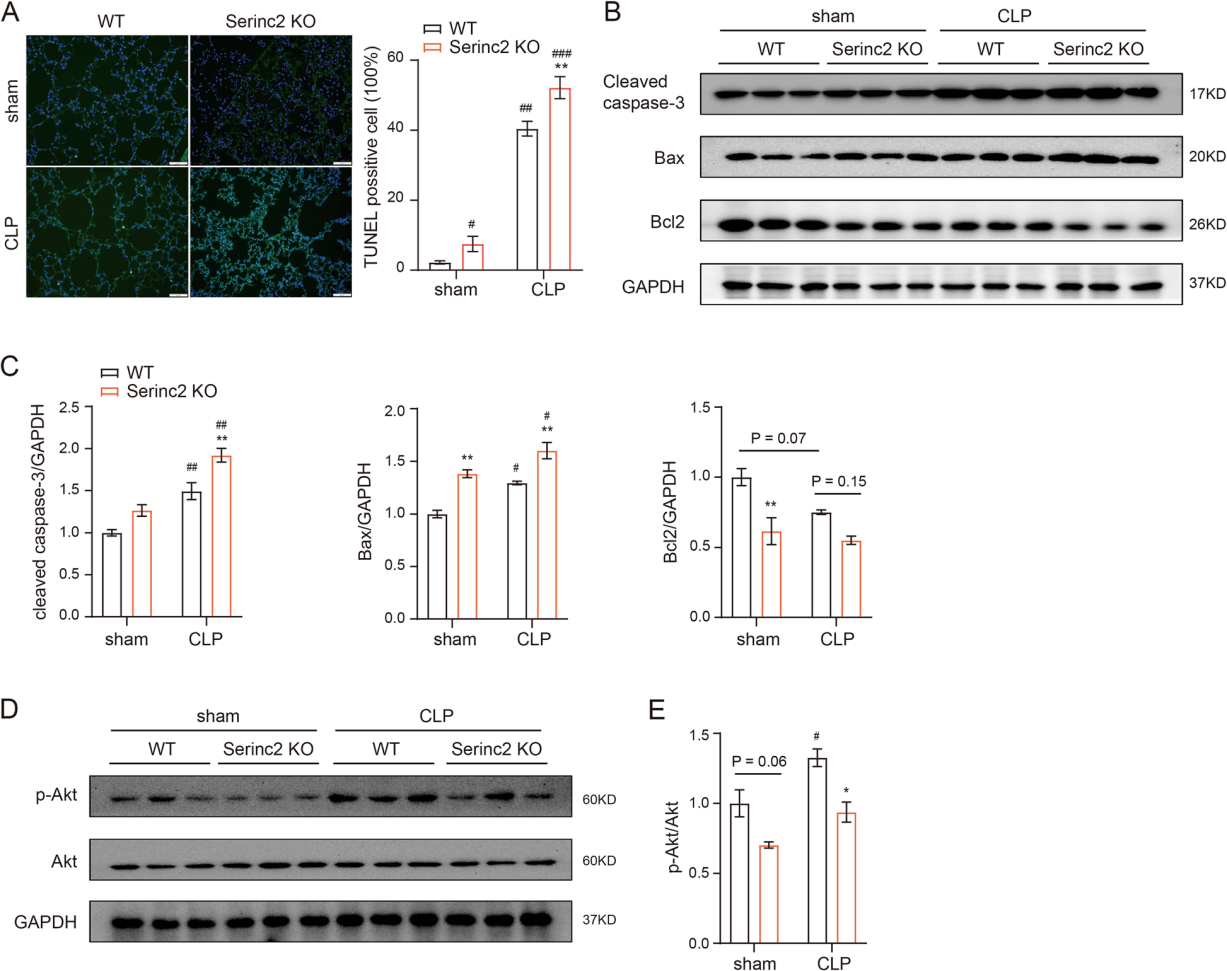
Serinc2 KO aggravates CLP-induced apoptosis in the lung. **A** Representative TUNEL staining images (left) and quantification data (right) showing the impact of Serinc2 KO on CLP-induced apoptosis. **P < 0.01 vs. WT; ###*P* < 0.001 vs. sham. *n* = 5. **B** Immunoblots showing the impact of Serinc2 KO on CLP-induced changes of cleaved caspase-3, Bax, and Bcl2. **C** Quantification of cleaved caspase-3, Bax and Bcl2 expression relative to GAPDH. **P < 0.01 vs. WT; #*P* < 0.05, ##*P* < 0.01 vs. sham. *n* = 3. **D** Immunoblots showing the impact of Serinc2 KO on CLP-induced Akt phosphorylation. **E** Quantification of Akt phosphorylation relative to its total protein. *P < 0.05 vs. WT; #*P* < 0.05 vs. sham. *n* = 3

Cell death is a major manifestation of ALI and plays a causal role in alveolar-capillary barrier damage, fluid leakage, and pulmonary hemorrhage [7–9]. We then examined the effect of Serinc2 KO on apoptosis by TUNEL staining of lung tissues with or without CLP surgery. The results showed that CLP significantly increased the number of TUNEL positive cells, which was further amplified in the Serinc2 KO group (Fig. 4A). Consistently, the increases in cleaved caspase-3 and Bax expression after CLP were enhanced by Serinc2 deficiency, while the reduced expression of Bcl2 was further diminished (Fig. 4B and C). AKT signaling pathway plays a pivotal role in cell survival [20, 21]. We observed that Akt phosphorylation was significantly activated by CLP, a pattern that was substantially reversed by Serinc2 KO (Fig. 4D and E). These data suggest that Serinc2 deficiency promotes apoptosis-associated cell death in ALI possibly dependent on Akt signaling pathway.

**Fig. 5:**
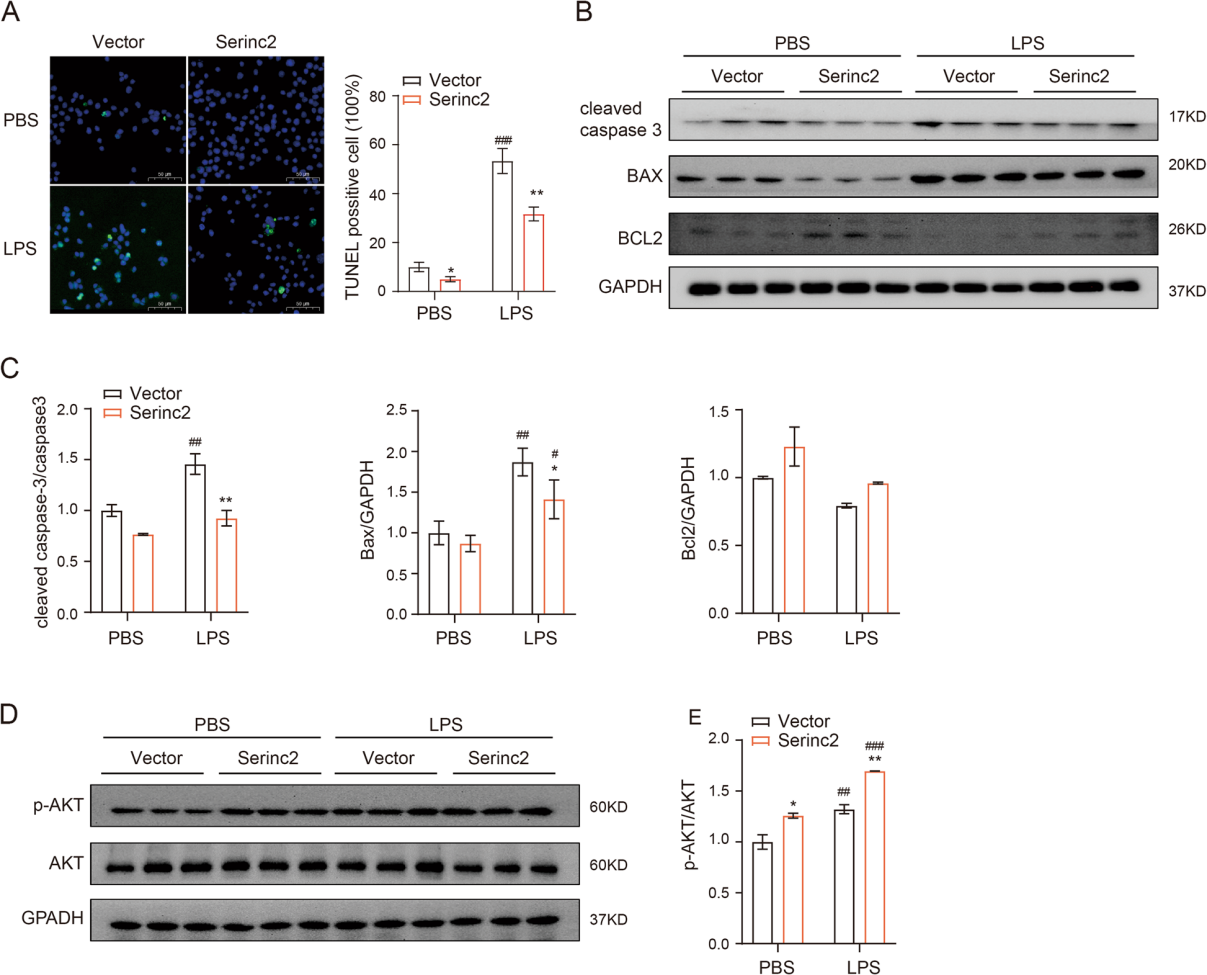
Serinc2 overexpression inhibits LPS-induced apoptosis in RAW264.7 cells. **A** Representative TUNEL staining images (left) and quantification data (right) showing the impact of Serinc2 overexpression on LPS-induced apoptosis in RAW264.7 cells. *P < 0.05, **P < 0.01 vs. Vector; ##*P* < 0.01 vs. PBS. *n* = 3. **B** Immunoblots showing the impact of Serinc2 overexpression on LPS-induced changes of cleaved caspase-3, Bax, and Bcl2. **C** Quantification of cleaved caspase-3, Bax and Bcl2 expression relative to GAPDH. *P < 0.05, **P < 0.01 vs. Vector; #*P* < 0.05, ##*P* < 0.01 vs. PBS. *n* = 3. **D** Immunoblots showing the impact of Serinc2 overexpression on LPS-induced Akt phosphorylation. **E** Quantification of Akt phosphorylation relative to its total protein. *P < 0.05, **P < 0.01 vs. Vector; ##*P* < 0.01, ###*P* < 0.001 vs. PBS. *n* = 3

To test the anti-apoptosis function of Serinc2, we examined the apoptosis levels in LPS-treated RAW264.7 cells with or without Serinc2 overexpression. The results showed that Serinc2 overexpression significantly suppressed the LPS-induced increase in TUNEL positive cells (Fig. 5A), and reversed the LPS-induced changes in cleaved caspase-3, Bax, and Bcl2 (Fig. 5B and C). Moreover, Serinc2 overexpression increased Akt phosphorylation at basal condition, and further enhanced it after LPS treatment (Fig. 5D and E). These results suggest a pro-survival role of Serinc2 in ALI.

### Akt signaling pathway mediates the pro-survival role of Serinc2 in ALI

**Fig. 6:**
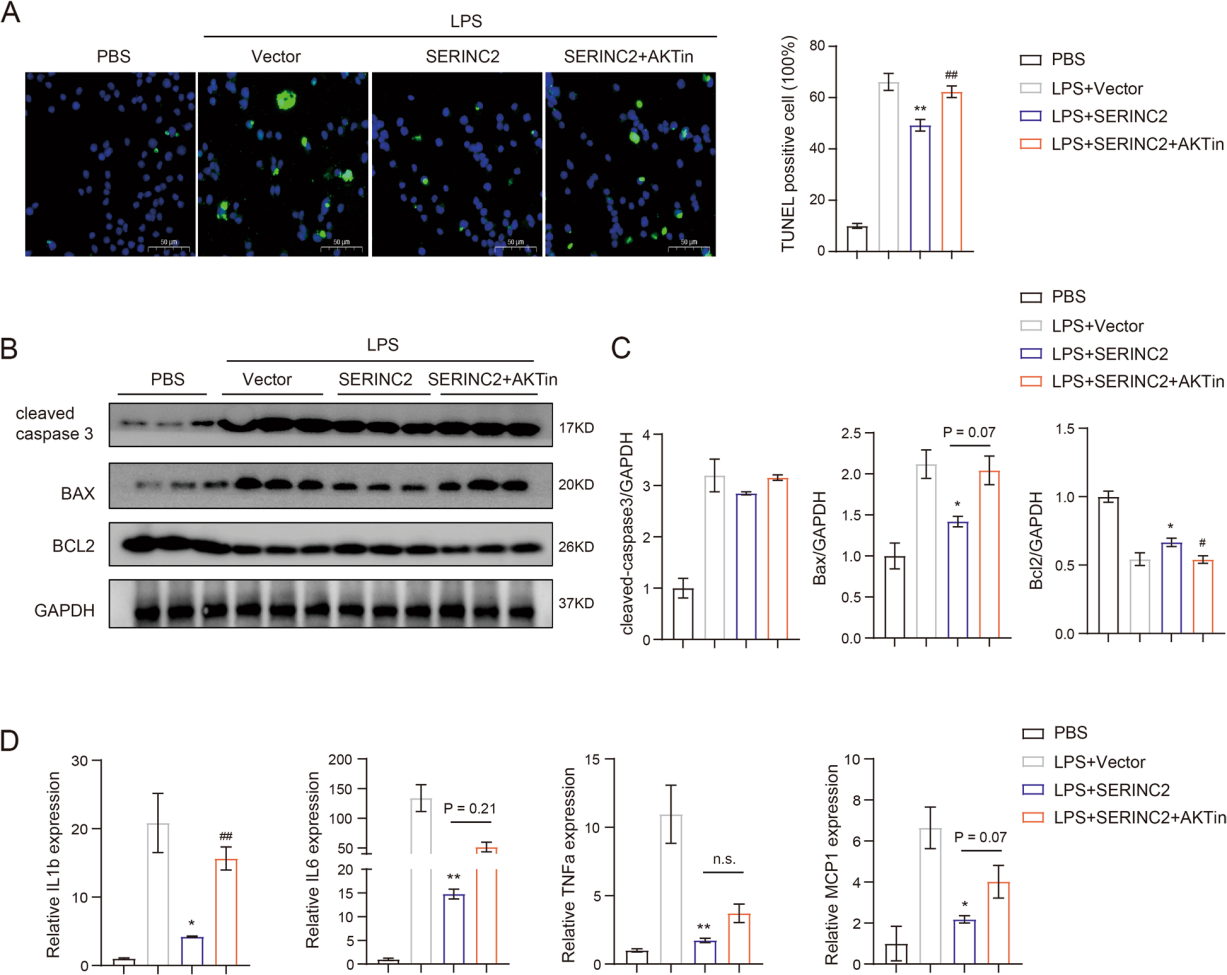
Akt pathway mediates the protective effects of Serinc2 in LPS-treated RAW264.7 cells. **A** Representative TUNEL staining images (left) and quantification data (right) in PBS, LPS + vector, LPS + Serinc2, and LPS + Serinc2 + AKTin groups. AKTin (1 μM) was administrated to RAW264.7 cells for 24 h. **P < 0.01 vs. LPS + Vector; ##*P* < 0.01 vs. LPS + Serinc2. *n* = 3. **B** Immunoblots of cleaved caspase-3, Bax, and Bcl2 in PBS, LPS + vector, LPS + Serinc2, and LPS + Serinc2 + AKTin groups. **C** Quantification of cleaved caspase-3, Bax and Bcl2 expression relative to GAPDH. *P < 0.05 vs. LPS + Vector; #*P* < 0.05, ##*P* < 0.01 vs. LPS + Serinc2. *n* = 3. **D** Expression of IL1β, IL6, TNFα, and MCP1 in PBS, LPS + vector, LPS + Serinc2, and LPS + Serinc2 + AKTin groups detected by qRT-PCR. *P < 0.05, **P < 0.01 vs. LPS + Vector; ##*P* < 0.01 vs. LPS + Serinc2. *n* = 3

To validate that Akt signaling pathway mediates Serinc2’s protective function in ALI, we employed an Akt-specific inhibitor AKTin [22–24]. Administration of AKTin (1 μM) in RAW264.7 cells significantly blocked the inhibition of LPS-induced increase in TUNEL positive cells by Serinc2 overexpression (Fig. 6A). Consistently, the protective effects of Serinc2 overexpression on LPS-induced increases in cleaved caspase-3 and Bax and decrease in Bcl2 were substantially reversed by AKTin treatment (Fig. 6B and C). Moreover, AKTin largely abolished the inhibition of Serinc2 overexpression on inflammatory factors, albeit not achieving statistical significance for IL6, TNFα, and MCP-1 (Fig. 6D). These data suggest that Akt signaling pathway contributes to Serinc2-mediated protection from apoptosis and inflammation.

## Discussion

The pathogenesis of sepsis-associated ALI is manifested by inflammatory cascade and apoptosis accumulation. In this study, we identify Serinc2 as a novel protective regulator in the development of ALI pathologies. The major findings include: (1) Serinc2 is dispensable for development and fertility; (2) Serinc2 expression is up-regulated in CLP-induced ALI; (3) Serinc2 plays a protective role in CLP-induced ALI both in vivo and in vitro; (4) Serinc2 suppresses hyperactive inflammation and excessive apoptosis; (6) AKT signaling pathway mediates the protective effects of Serinc2.

The Serinc family contains five members: Serinc1, Serinc2, Serinc3, Seinc4, and Serinc5. By integrating serine, a non-essential amino acid, into the cell membrane, its crucial function is to promote the production of phosphatidylserine and sphingomyelin and regulate the biosynthesis of membrane lipid molecules [11]. The research on the interaction mechanism between virus and host cell has always been a research hotspot and is of important scientific significance. As an important antiviral protein family discovered in recent years, the Serinc family has attracted more and more attention from many scholars. Serinc5 has been proved to be the most powerful antiviral factor and can inhibit HIV, MLV, EIAV, and so on [25]. It selectively inactivates envelope glycoprotein, interferes with the remaining env activity, prevents the folding of the envelope, and then limits the infection of offspring virus to new target cells [26]. In addition, serinc1 and serinc3 have also been observed to have limited HIV inhibition. Interestingly, Serinc2, also known as tumor differentially expressed 2 (TDE2), has been firstly identified in non-small cell lung cancer cells [12] and the carcinogenic transformation of the tumor was often accompanied by the high expression of Serinc2 or its family members, and it was further found that it had no effects on the infectivity of HIV-1 [26]. Here we, for the first time, reveal a protective role of Serinc2 in sepsis-associated ALI through inhibiting apoptosis and inflammation. And we infer that Serinc2 can participate in many life processes and affect the progression of different diseases. Its antiviral effect in the process of virus infection and its antagonism with virus proteins have great research potential.

The plasma membrane is of great significance for the extracellular to intracellular transmembrane signal transduction of membrane receptors. Plasma membrane microcapsules and lipid rafts are lipid-ordered domains rich in cholesterol and sphingomyelin and enrich a variety of intracellular proteins involved in signal transduction [27]. Intact plasma membrane microcapsules and lipid rafts are the important molecular basis for signal transduction, especially transmembrane signal transduction [27]. Emerging evidence reveals that the production of phosphatidylserine, “apoptosis clearance signaling”, inhibits leukocyte migration and promotes inflammation resolution [28]. As a cofactor, phosphatidylserine is required for the activation of the TAM family, and further plays a counter-inflammation response in macrophages [29, 30]. The main function of Serinc2 is to assist in lipid synthesis on plasma membrane [11]. Our findings indicate that Serinc2 might contribute to the quench of the activated inflammation signaling, which depends on lipid raft integrity and mobility. As a key molecule in the synthesis of membrane lipids, whether Serinc2 also plays a protective role in ALI via the synthesis of phosphatidylserine and sphingomyelin needs to be investigated in future.

Production of inflammatory factors is mainly mediated by transcription factors, especially STAT3, which is responsible for transducing extracellular stimuli to nuclear gene expression through translocation from cytosol to nucleus after phosphorylation [31]. The activation of the STAT3 signaling pathway is closely related to the occurrence of a variety of inflammatory diseases, such as ulcerative colitis [32], rheumatoid arthritis [33], and sepsis [34], especially in the sepsis-mediated inflammatory reaction. This signaling pathway has an important impact on the production of proinflammatory factors such as interferon and TNFα. Wang et al. [34] used lipopolysaccharide to induce acute lung injury in rats and found that inflammatory cytokines such as IL6 and IL1 were up-regulated in serum and the expression of the p-STAT3 was much more up-regulated compared to the sham group. Persistent activation of STAT3 mediates both the release of proinflammatory cytokines and the suppression of anti-immune response in M1 pro-inflammatory macrophages [35–37]. This was further confirmed by our findings that the increasing phosphorylation lever of STAT3 according to time the of LPS challenge and the expression of p-STAT3 was downregulated while the inflammatory response was alleviated.

As the center of signal transduction pathways, MAPK (mitogen-activated protein kinase, MAPK) pathway can be activated by various stimuli such as cytokines, radiation, osmotic pressure [38]. Activated MAPK receives signals that are converted and transmitted by membrane receptors and carries them into the nucleus, playing a pivotal role in the production of inflammatory cytokines and other bio functions. ERK1/2, which widely exists in various tissues, is involved in cell proliferation and differentiation. It is found that LPS can bind to Na–H exchanger 1 of rat alveolar cell membrane, activate macrophages and endothelial cells through ERK1/2 phosphorylation, thus produce inflammatory factors such as IL-8, TNFα, and MIP2 and cause pulmonary dysfunction [39]. As the most important member of the MAPK signaling family to regulate the inflammatory response, the p38 significantly promotes the development of inflammation in the process of ALI. Studies have shown that in the process of ALI, activated p38 significantly increases the expression of IL6 and IL1, the following respiratory burst leading to the increase of pulmonary endothelial cell permeability and the formation of pulmonary edema pathologically. Many p38 specific inhibitors have shown early anti-inflammatory efficacy, even the p38γ inhibitor has been used in the clinical treatment of idiopathic pulmonary fibrosis [18, 40]. In the present study, we found that p38 and ERK were evoked by inflammatory stimuli including CLP and LPS, which could be modified by Serinc2. Considering the function of Serinc2 in membrane trafficking, our data implicate a crucial role of Serinc2-mediated membrane event in organizing transmembrane signaling transduction in the lung. Sepsis caused by LPS can not only damage the lung but also damage the functions of the heart, liver, and kidney. Whether Serinc2 can inhibit the acute injury of the heart, liver, and kidney caused by LPS and play a protective role in addition to the lung remains unexplored. Further investigations are required to explore the function of Serinc2 in these systems, as well as the molecular mechanisms.

The activation of AKT mainly occurs on the cell membrane. When cells suffered from extracellular signals, activated PI3K generates PIP3 and transposes it to the cell membrane, which not only enables AKT itself to obtain catalytic activity but also enables Both AKT and PDK-1 to be co-located on the cell membrane. PDK-1 can further catalyze AKT phosphorylation and make it fully activated, thereby regulating the phenotypes of cell proliferation, differentiation, apoptosis, and migration [23, 41]. Corresponding to in vivo experiments, we used an inflammatory response model of ALI induced by LPS. It was confirmed that up-regulation of Serinc2 can lead to a decrease in the apoptosis-associated protein and reduction of TUNEL cells. Furthermore, our in vitro analyses further verify that Serinc2 could inhibit apoptosis by activation of Akt, as evidenced by negligible changes in LPS-induced effects, containing elevated cleaved-caspase3, Bax, and decreased Bcl2 protein expression, along with increases in TUNEL cells after AKTin pre-treatment. Additionally, others demonstrate activation of the PI3K/Akt signaling pathway suppresses the LPS-induced inflammatory caspases in ALI [42, 43]. In the present study, we found the inhibitor of Akt blocked the protective effect of Serinc2 against inflammation in ALI, implying that Serinc2 prevents LPS-induced inflammation and apoptosis partially by the Akt pathway. Previous studies have shown that Serinc2 can inhibit the development of the lung adenocarcinoma through PI3K / Akt signaling pathway, and for the first time, we elaborated the protective effect of Serinc2 in acute lung injury from the perspective of Akt mediated apoptosis and inflammation. However, it is unclear whether the special membrane trafficking function of Serinc2 can activate Akt or there is a spatial interaction between Serinc2 and Akt, which needs further experimental proof.

COVID-19 pandemic and its subsequent development into ALI/ARDS threaten the lives of millions of people worldwide, and the adverse progress of COVID-19 is related to the severe and aberrant inflammatory response and cell injury by virus infection. COVID-19 mainly invades the respiratory tract. On the one hand, viruses replicate in respiratory epithelial cells and vascular endothelial cells and damage lung tissue. On the other hand, viruses activate monocytes/macrophages, neutrophils, lymphocytes, and endothelial cells, releasing a mounting number of cytokines and chemokines, such as TNFα and IL6, causing a series of cytokine storms, leading to acute lung injury. This is similar to the characteristic of the acute lung injury caused by LPS. According to our above research results, Serinc2 can dampen the inflammatory reaction and apoptosis in the process of LPS induced acute lung injury, and may also participate in the acute lung injury caused by virus infection and play a protective role. As a positive participant of endogenous protective feedback response, Serinc2 can not only regulate inflammatory signal pathways, promote cell survival and inhibit cell apoptosis, but also can be a promising candidate in the development of new anti-COVID-19 approaches and may be an effective target to treat COVID-19 clinical complications.

## Conclusion

Taken together, our data demonstrate that Serinc2 functions as an endogenous protector against sepsis-associated ALI through suppression of STAT3, p38, and ERK pathways and activation of the Akt pathway. Our findings provide novel insights into the pathogenesis of sepsis-associated ALI and reveal potential strategies to treat ALI in the clinic.

## Supplementary Information

**Additional file 1.**

**Additional file 2.**

## Data Availability

All data and materials in this manuscript are available via contacting the corresponding author on proper request. GEO databases are available online (https://www.ncbi.nlm.nih.gov/).

## Abbreviations

AKT: Protein kinase B
ALI: Acute lung injury
ARDS: Acute respiratory distress syndrome
CLP: Cecal ligation and puncture
CXCL2: CXC chemokine ligands 2
EB: Evans blue
EIAV: Equine infectious anemia virus
ELISA: Enzyme-linked immunosorbent assay
ERK: Extracellular signal-regulated kinase
GEO: Gene Expression Omnibus
H&E: Hematoxylin and Eosin
HIV: Human immunodeficiency virus
IL1: Interleukin-1
IL6: Interleukin-6
IL8: Interleukin-8
KO: Knockout
LPS: Lipopolysaccharide
MAPK: Mitogen-activated protein kinase
MCP-1: Monocyte chemoattractant protein-1
MIP2: Macrophage inflammatory protein-2
MLV: Murine Leukemia Virus
Serinc2: Serine incorporator 2
STAT3: Signal transducers and activators of transcription 3
TNFα: Tumor necrosis factor α
TUNEL: Terminal deoxynucleotidyl transferase dUTP nick end labeling
