# Supplementary material for: Serinc2 deficiency causes susceptibility to sepsis-associated acute lung injury
Source: J Inflamm (Lond). 2022 Jul 7;19:9. doi: 10.1186/s12950-022-00306-x (PMC9260995; doi:10.1186/s12950-022-00306-x)
Supplement: Supplementary file 1 — Additional file 1. [file 12950_2022_306_MOESM1_ESM.docx]

**Supplementary Table 1. Serinc2 KO mice have normal mendelian fertility.**

| Genotype | Mendelian Expected | Actual Number (male/female) |
| --- | --- | --- |
| serinc2^-/-^ | 15 | 9 (4/5) |
| serinc2^+/-^ | 30 | 39 (19/20) |
| serinc2^+/+^ | 15 | 12 (9/3) |
| total | 60 | 60 (32/28) |
