# Supplementary material for: Serinc2 deficiency causes susceptibility to sepsis-associated acute lung injury
Source: J Inflamm (Lond). 2022 Jul 7;19:9. doi: 10.1186/s12950-022-00306-x (PMC9260995; doi:10.1186/s12950-022-00306-x)

Figure 1C

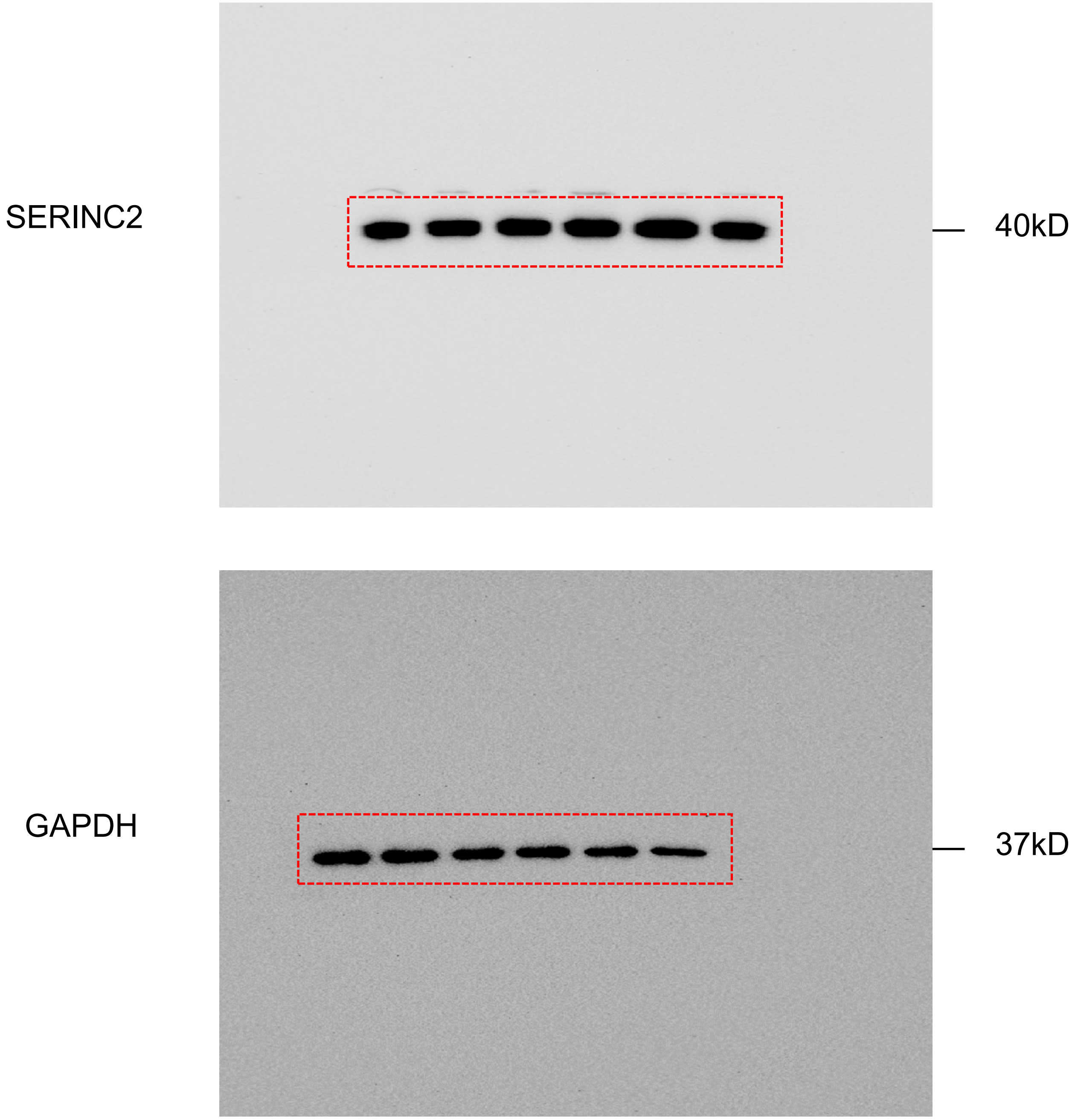

Figure 1F

SERINC2

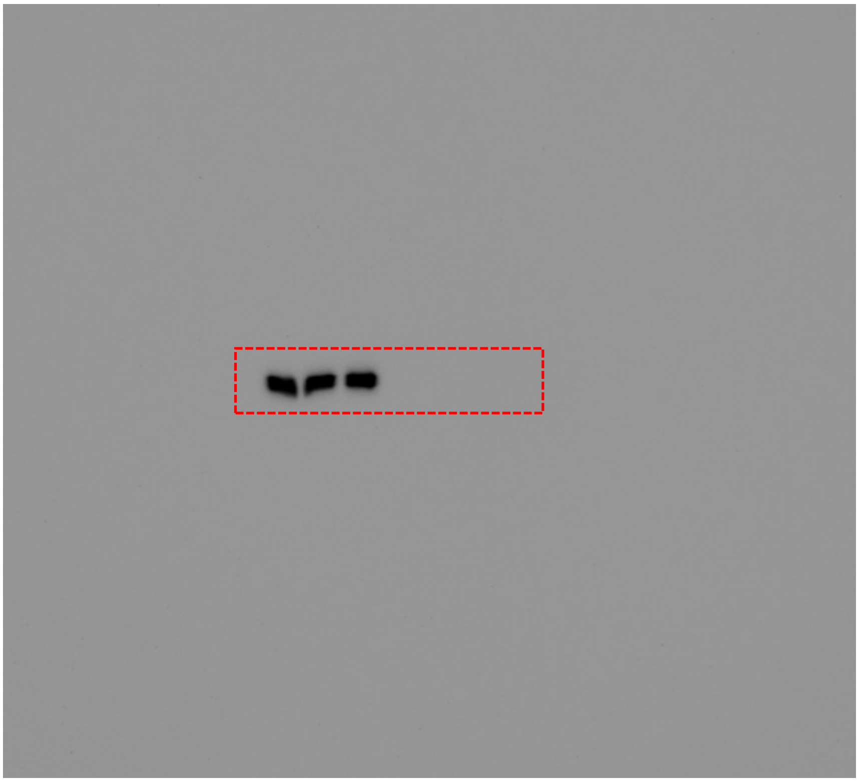

40kD

GAPDH

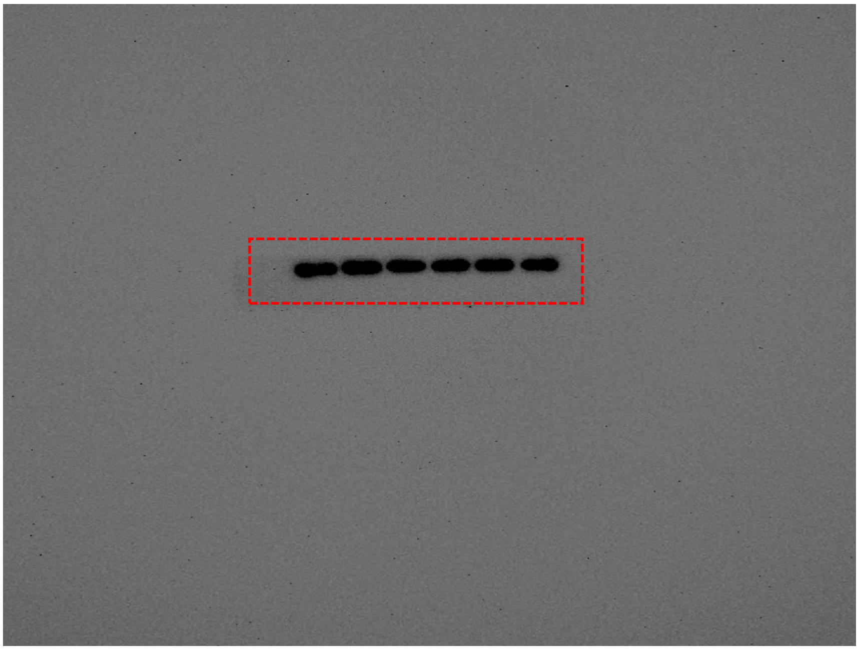

37kD

Figure 2C

p-stat3

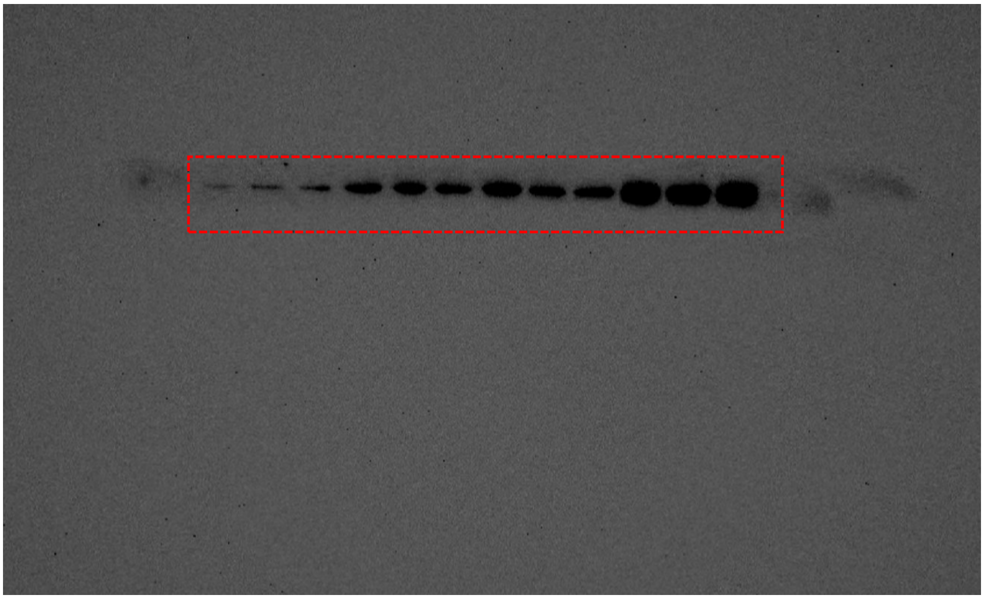

86KD

stat3

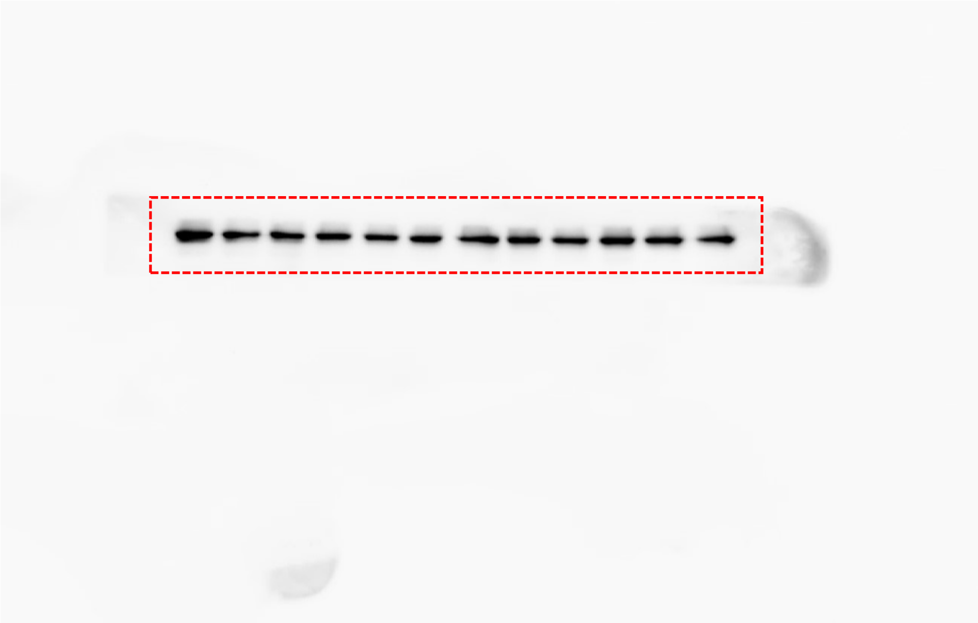

86KD

Figure 2C

p-P38

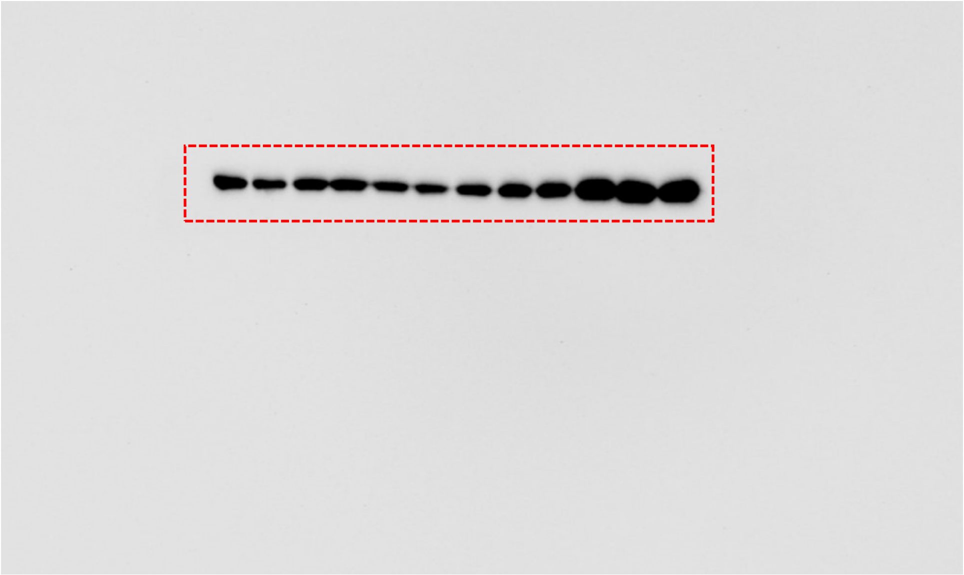

38KD

P38

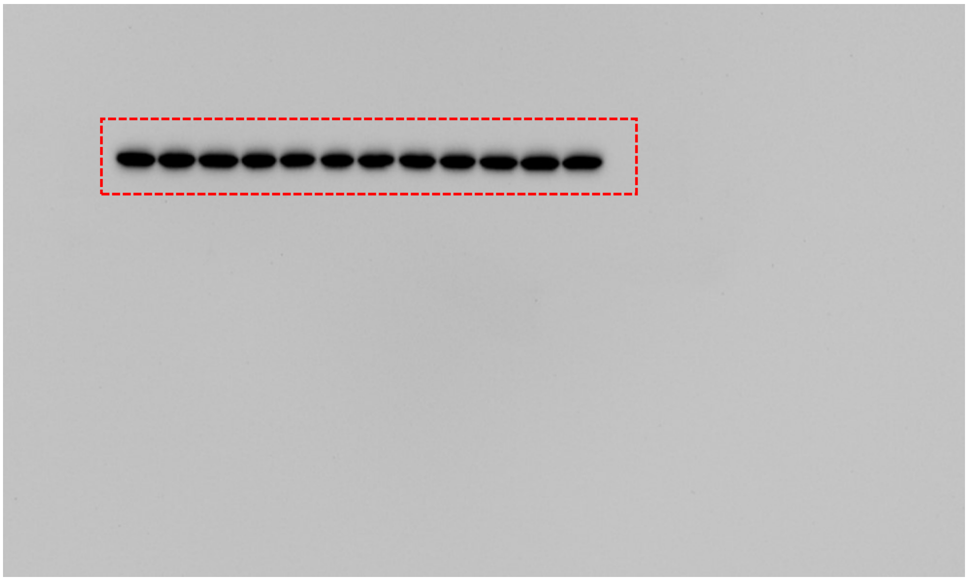

38KD

Figure 2C

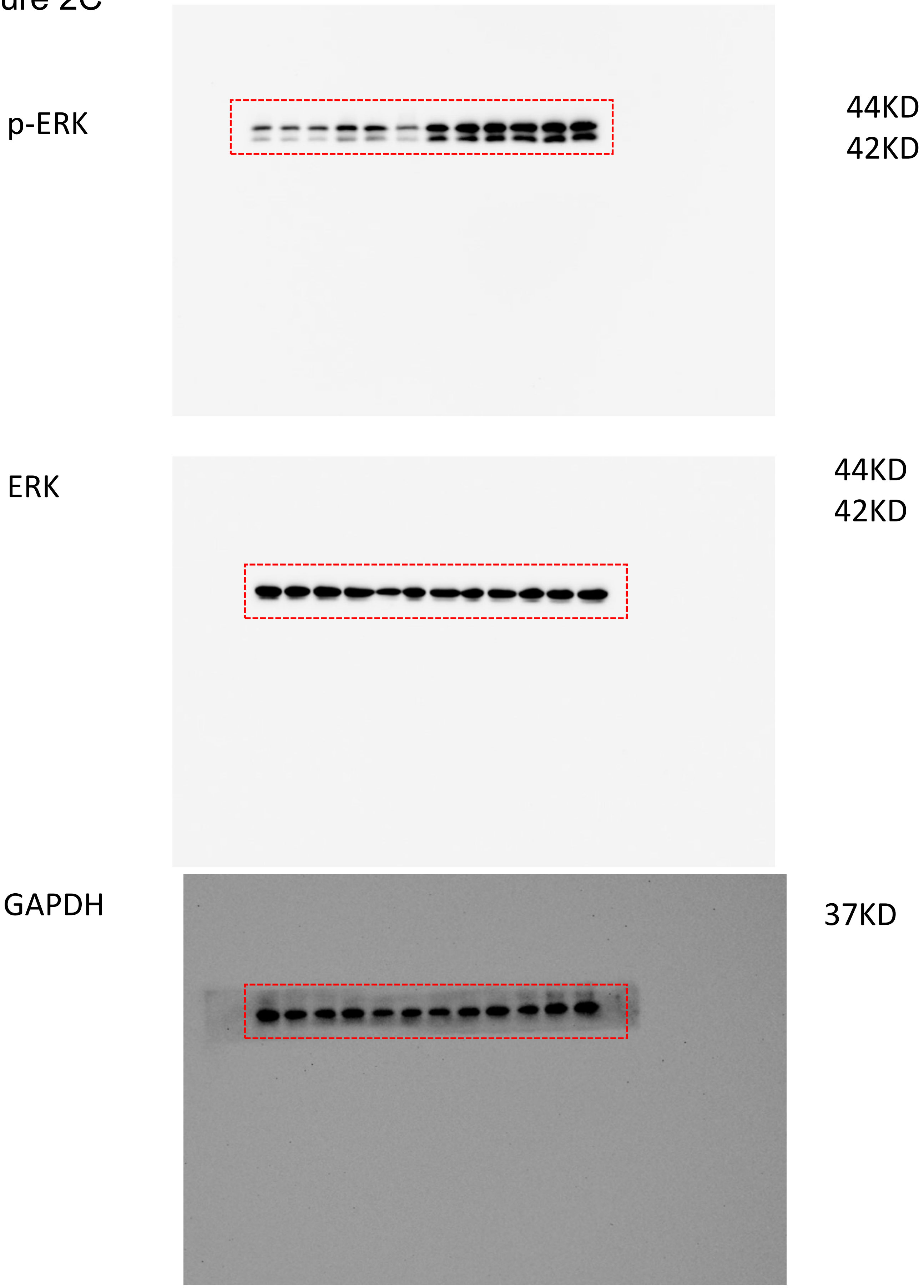

Figure 3C

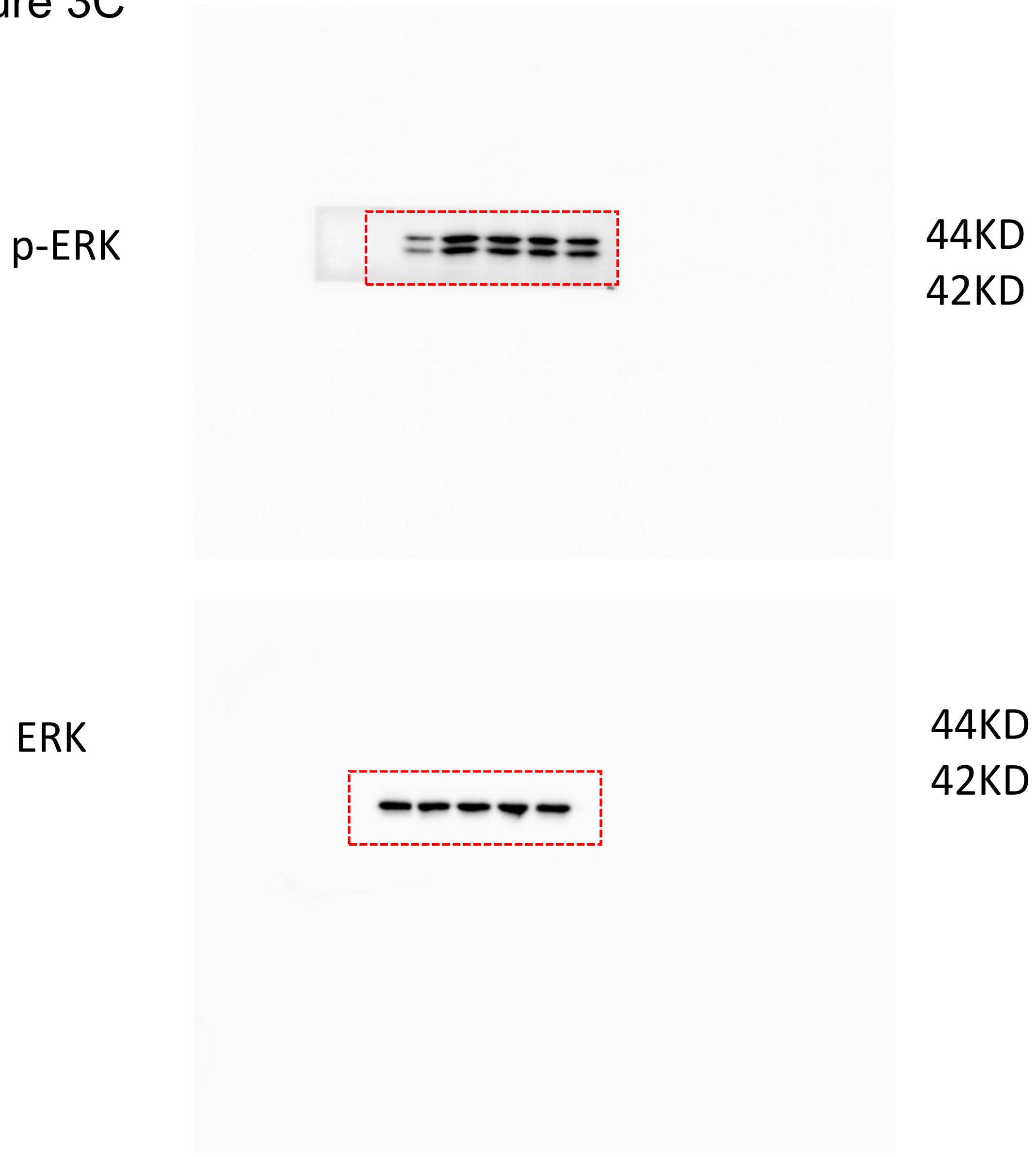

Figure 3C

p-p38

38KD

p38

38KD

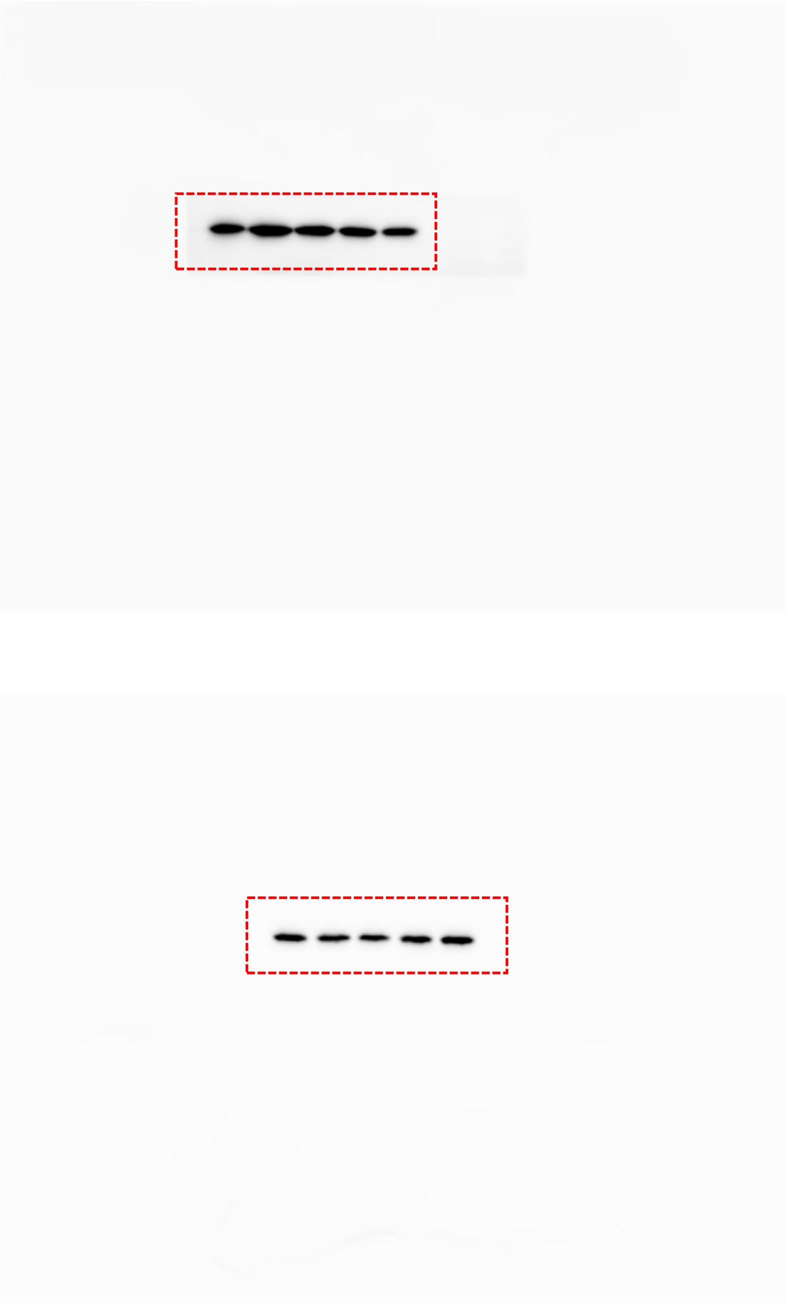

Figure 3C

p-stat3

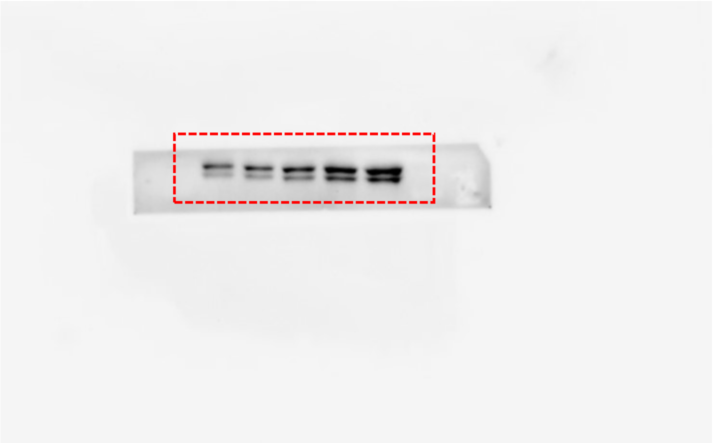

86KD

stat3

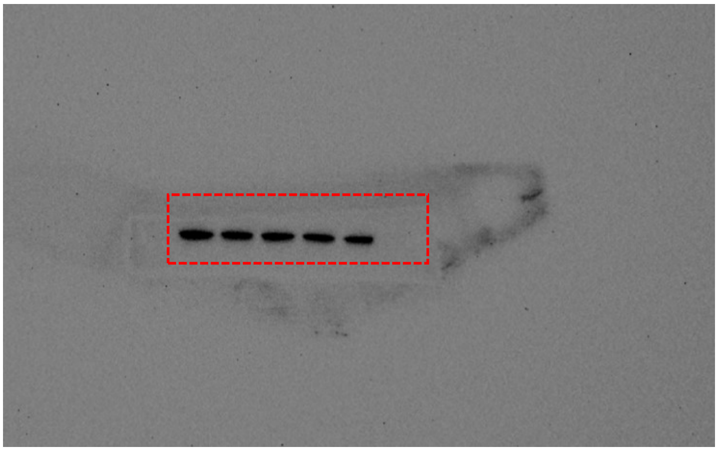

86KD

GAPDH

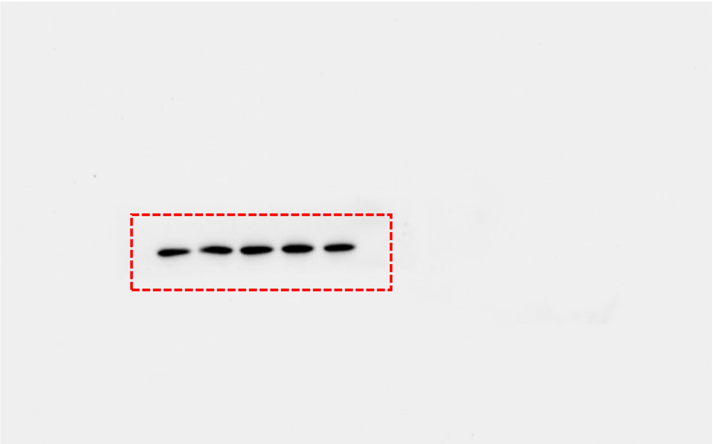

37KD

Figure 3D

p-stat3

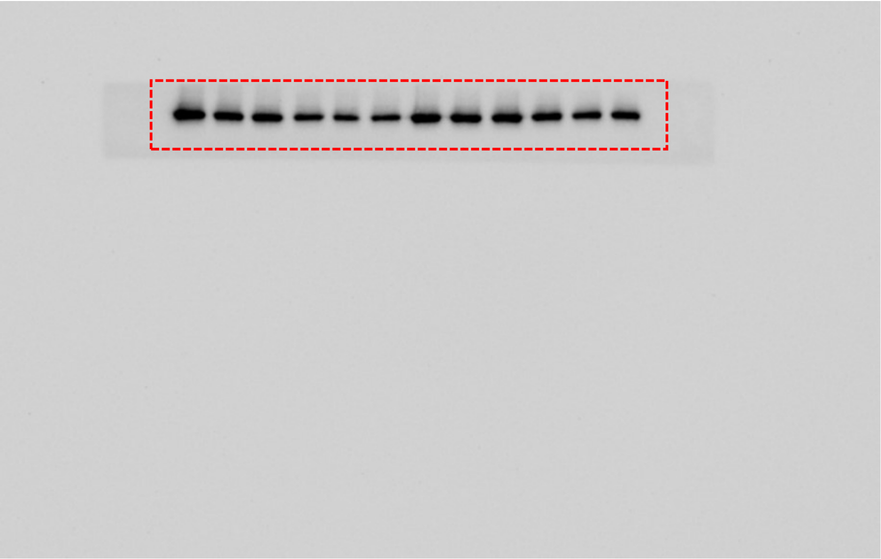

86KD

stat3

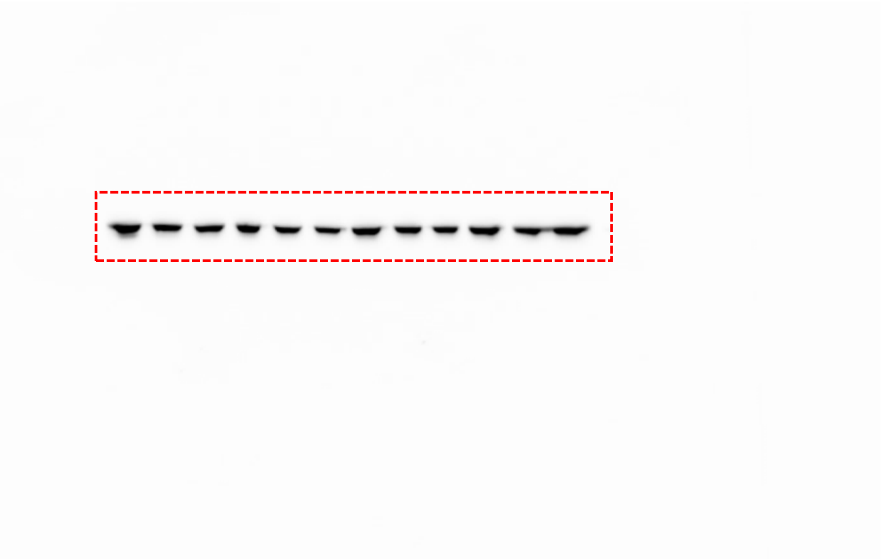

86KD

Figure 3D

p-p38 38KD

p38 38KD

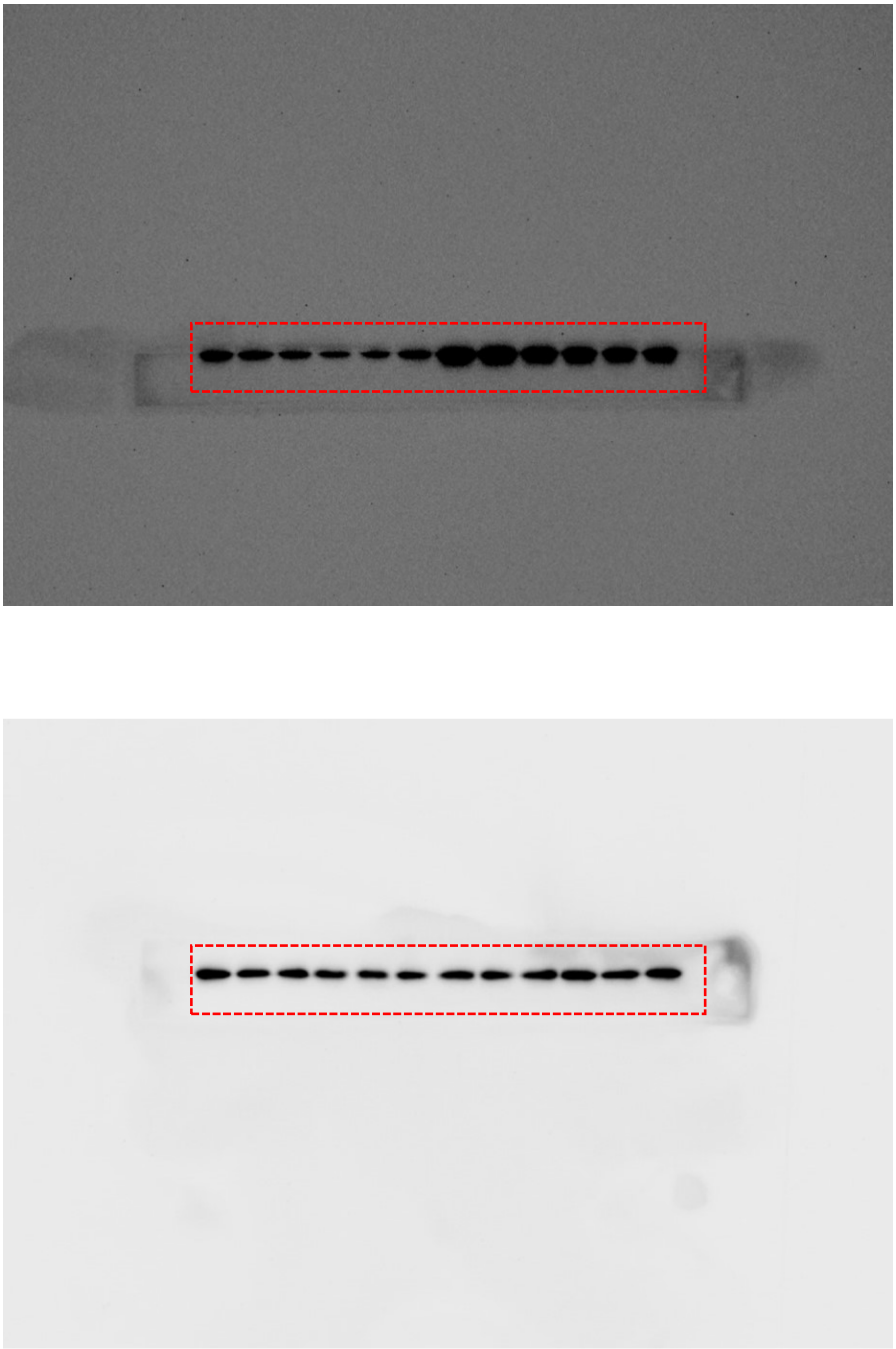

Figure 3D

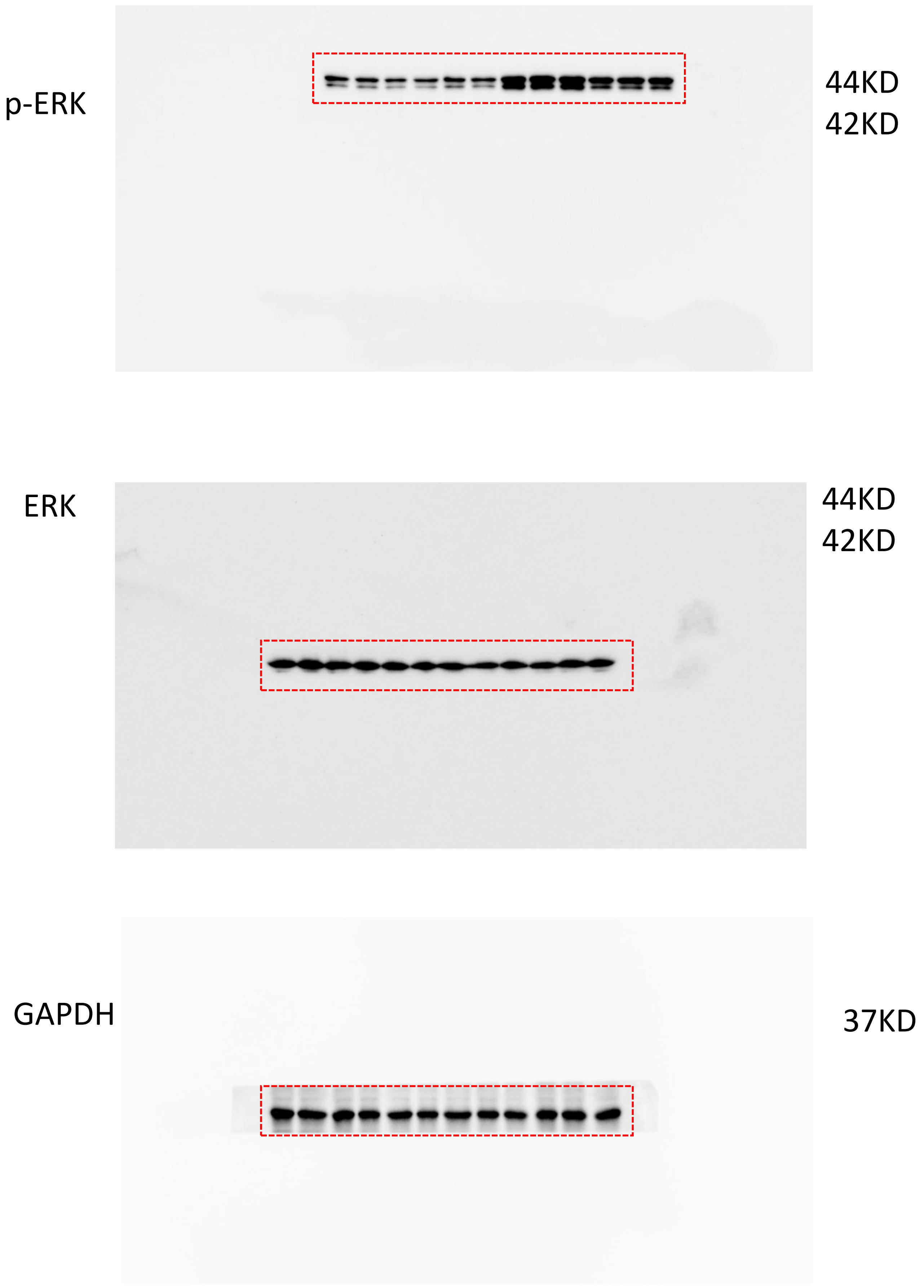

Figure 4B

Cleaved caspase3

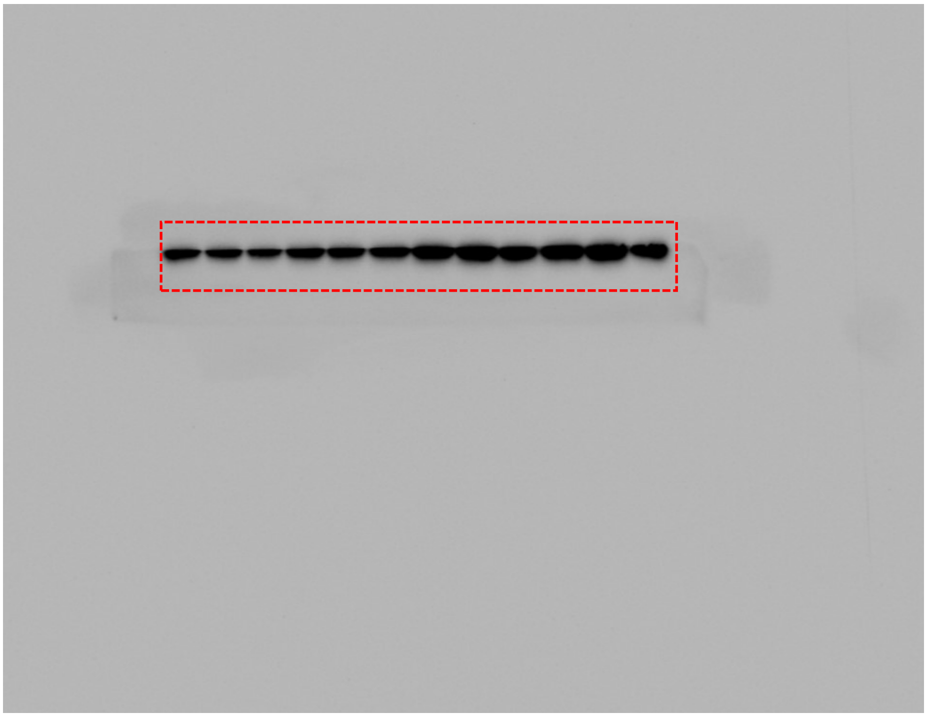

19KD

BCL2

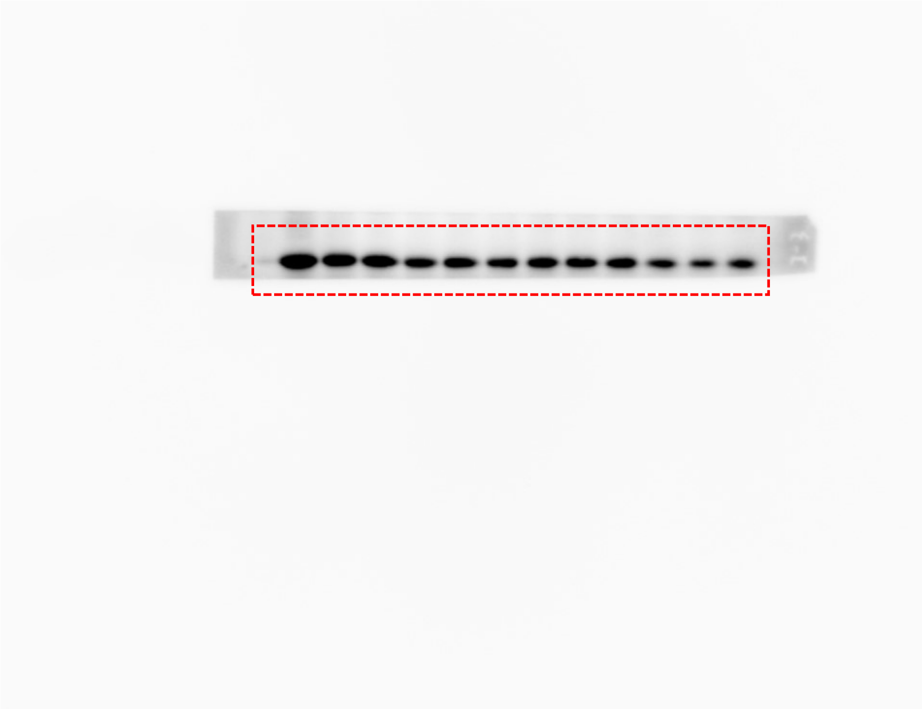

26KD

Figure 4B

Bax

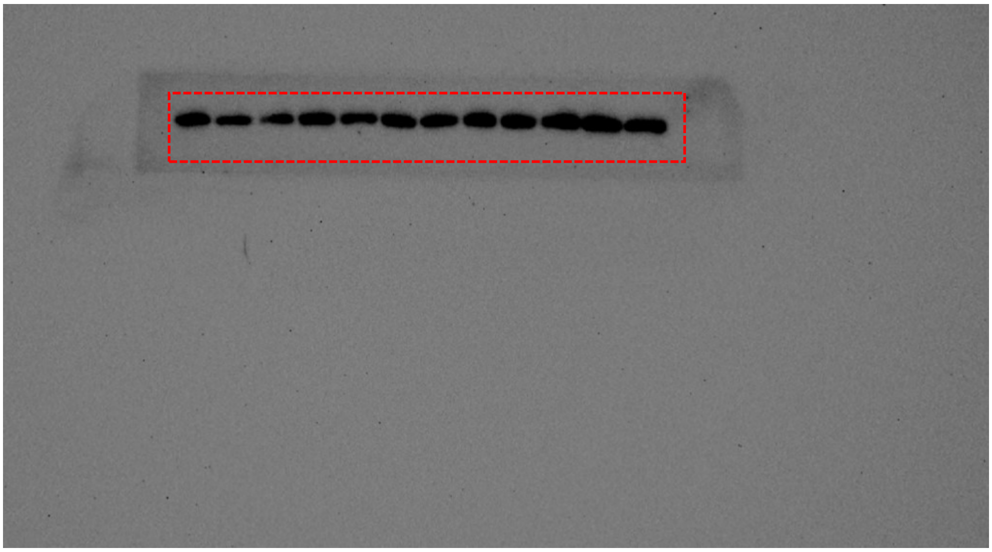

20KD

GAPDH

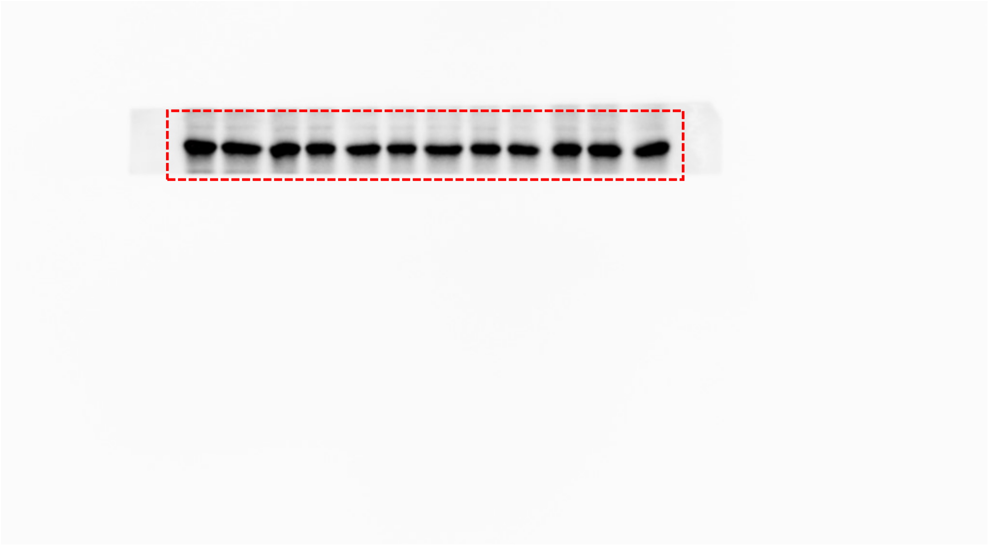

37KD

Figure 4D

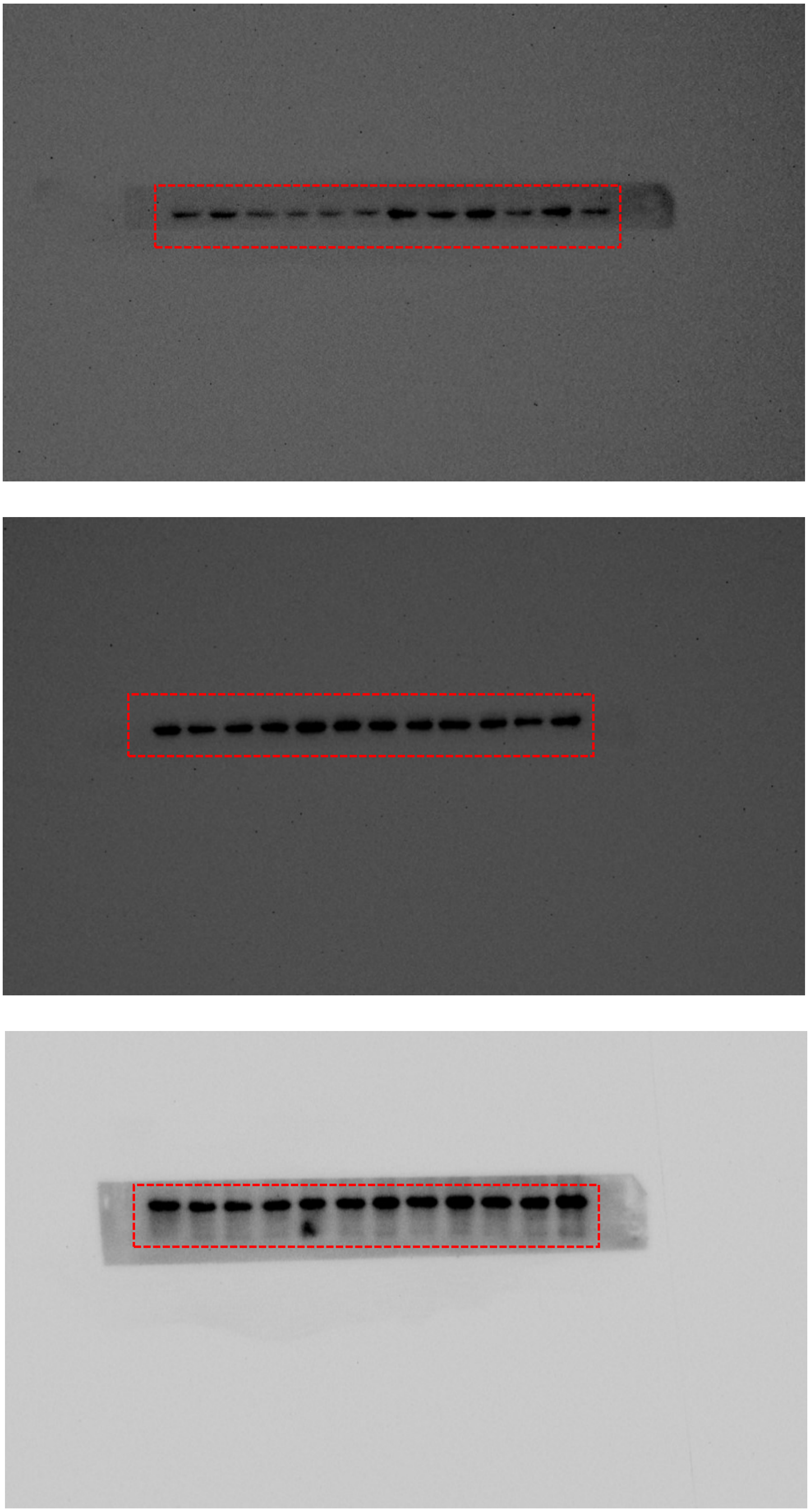

Figure 5B

Cleaved caspase3

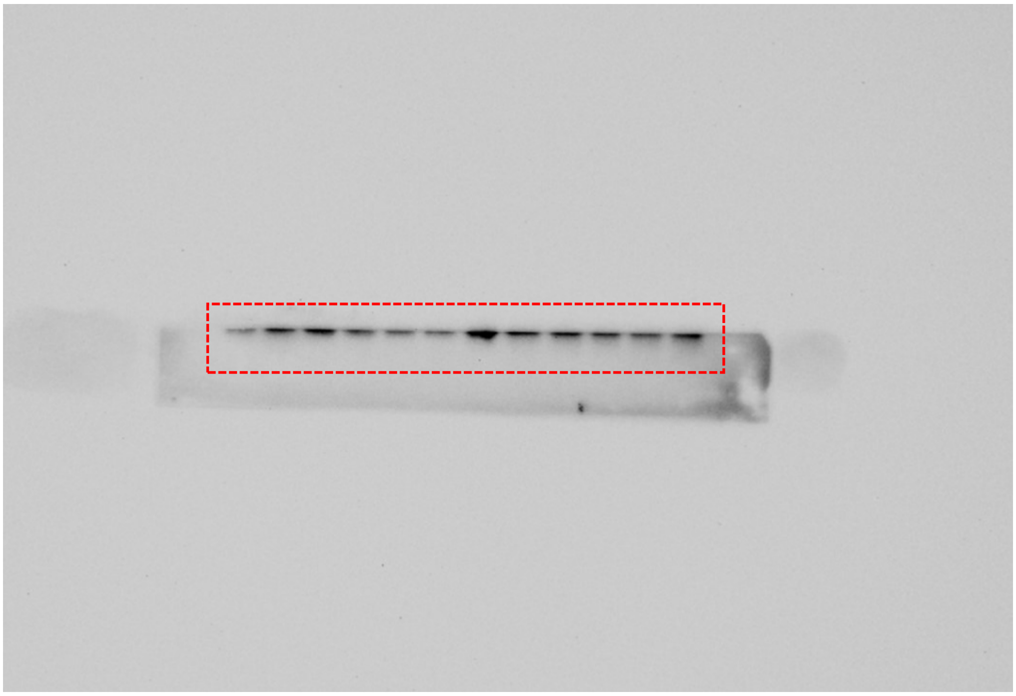

19KD

BAX

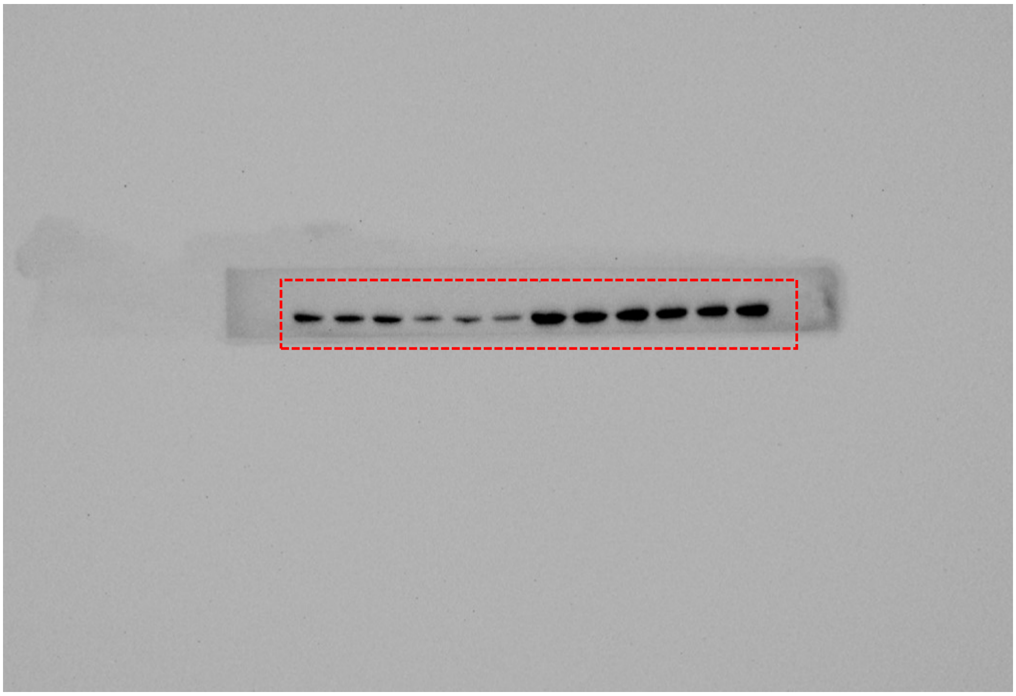

20KD

Figure 5B

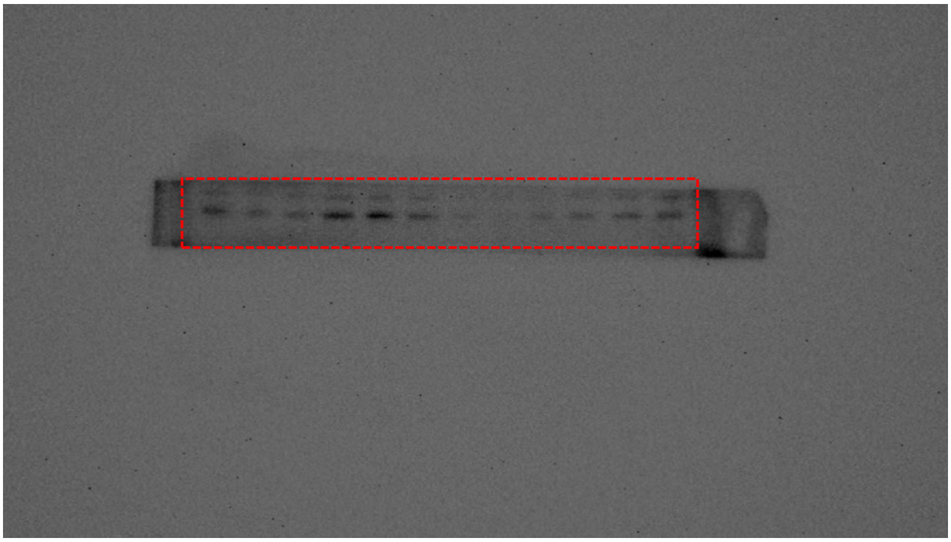

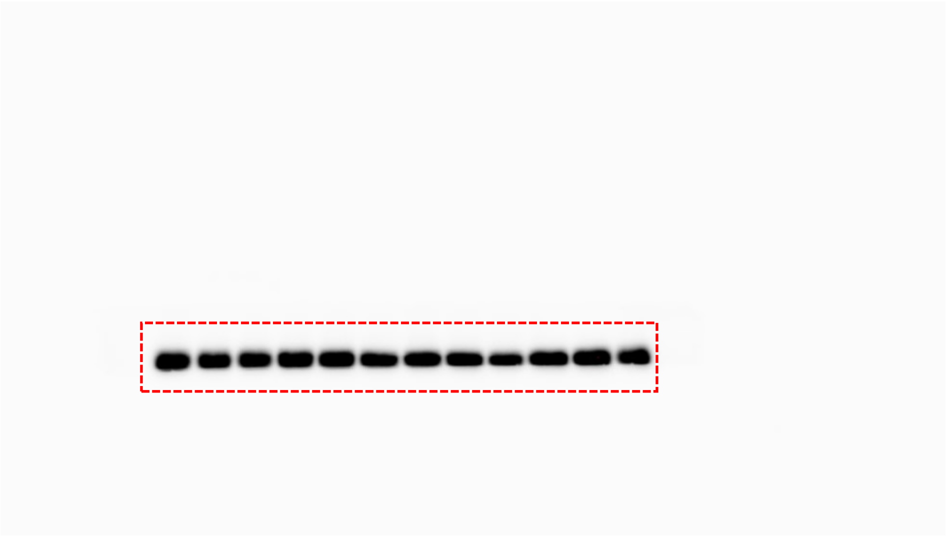

Figure 5D

P-AKT

AKT

GAPDH

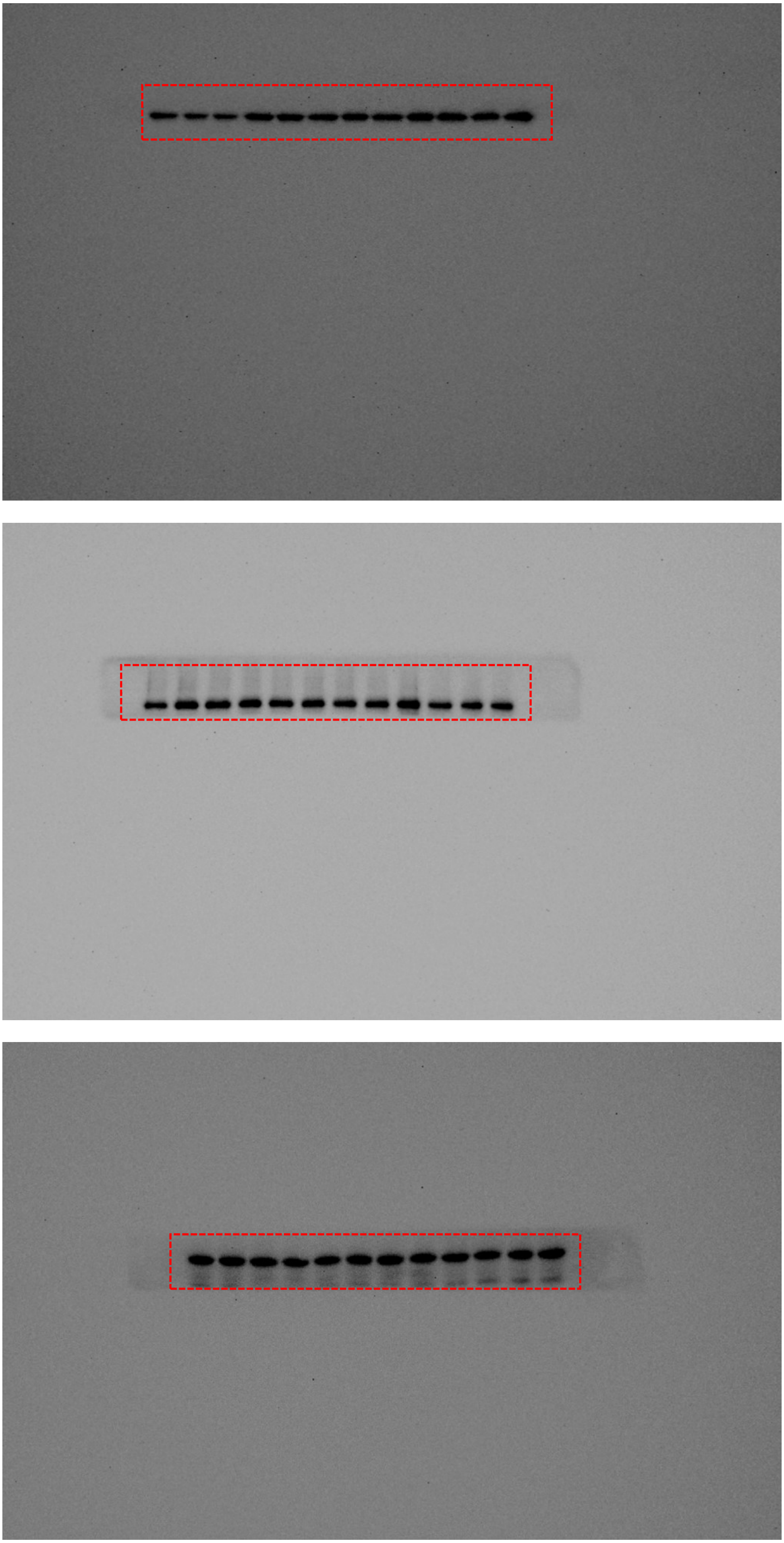

60KD

60KD

37KD

Figure 6B

Cleaved caspase 3

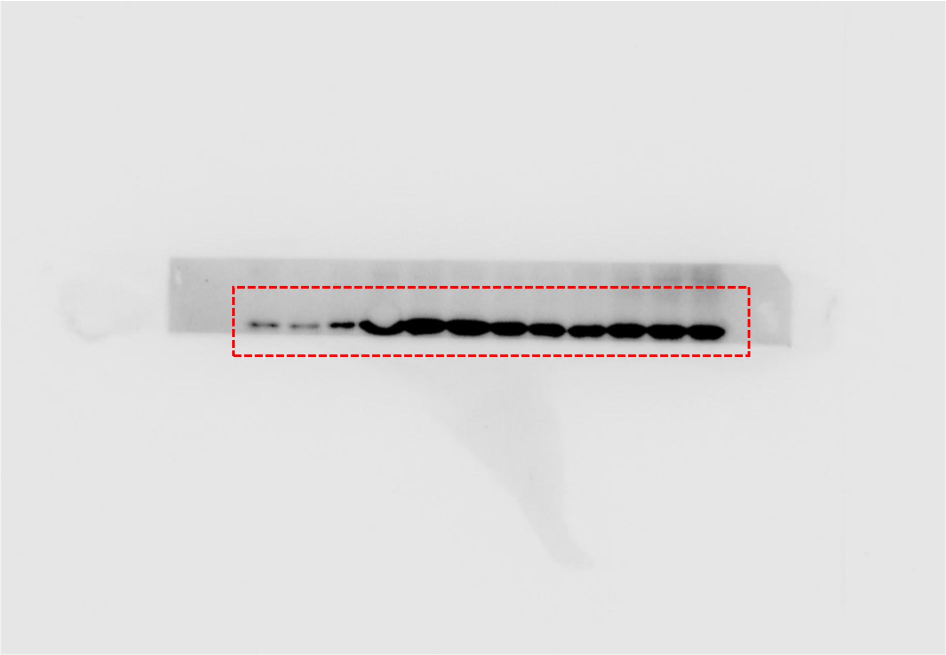

19KD

BAX

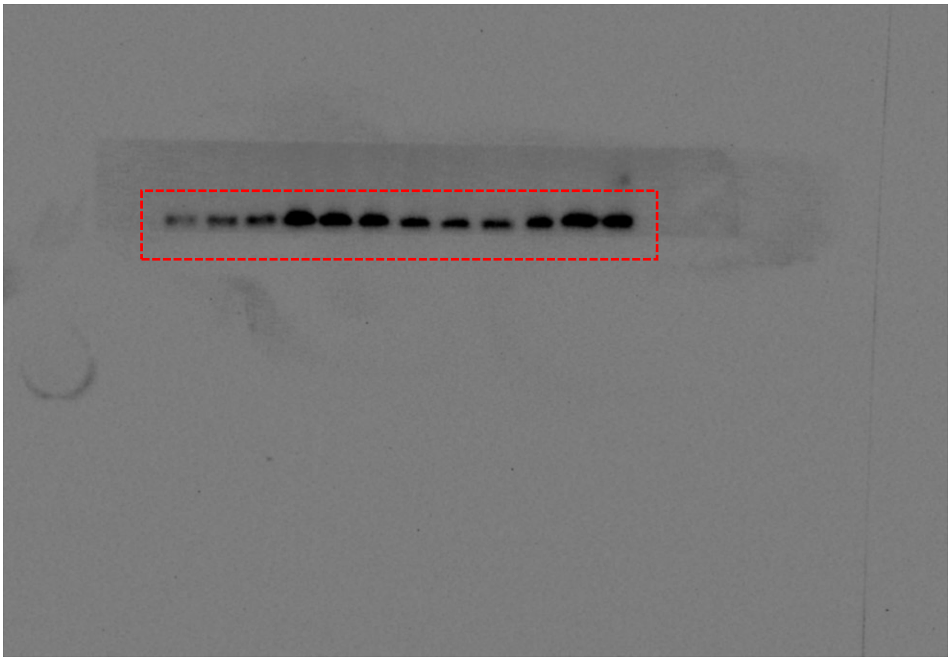

20KD

Figure 6B

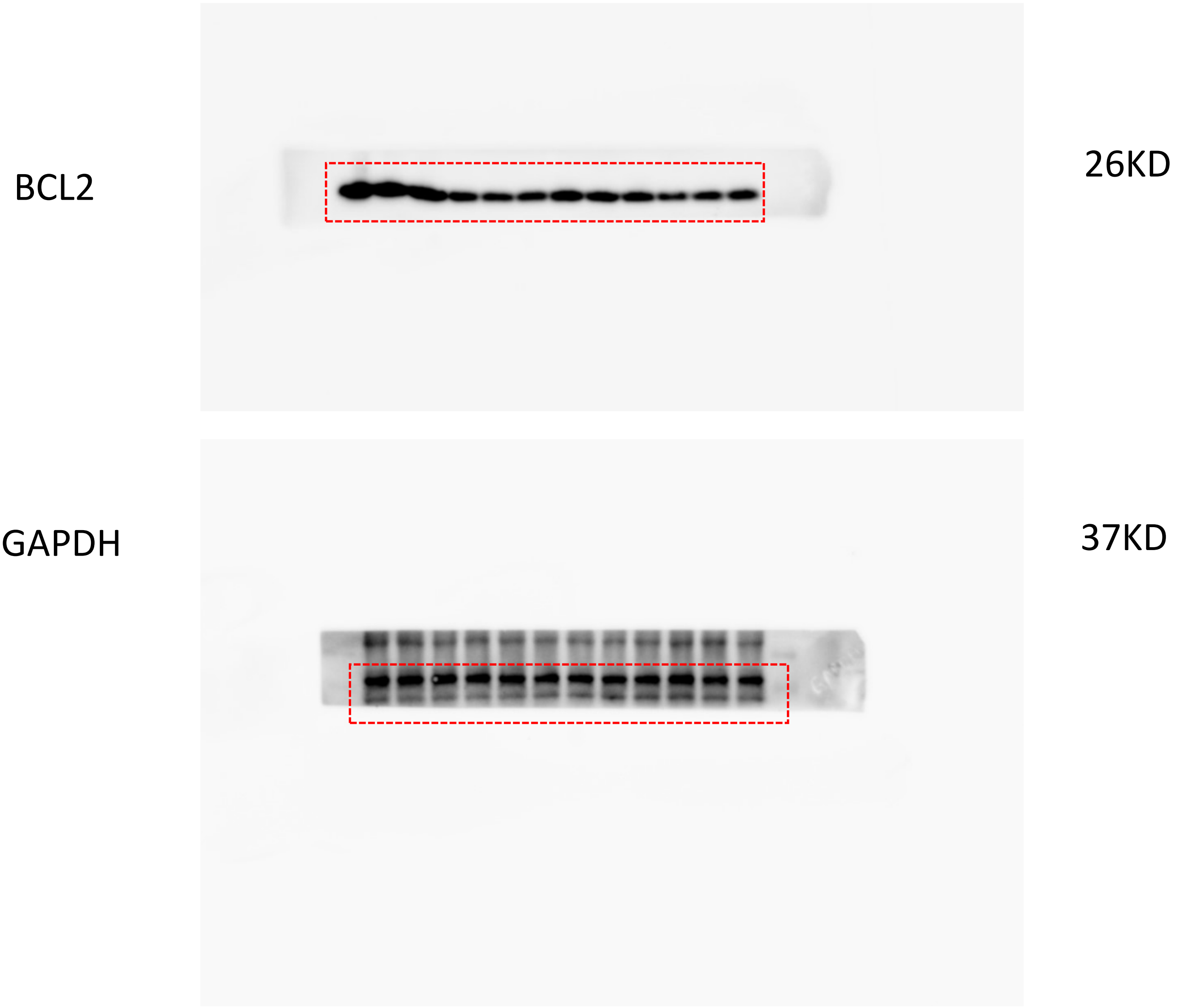

Supplement: Supplementary file 2 — Additional file 2. [file 12950_2022_306_MOESM2_ESM.pdf]
